# Long-term seed burial reveals differences in the seed-banking strategies of naturalized and invasive alien herbs

**DOI:** 10.1038/s41598-022-12884-0

**Published:** 2022-05-25

**Authors:** Lenka Moravcová, Angelino Carta, Petr Pyšek, Hana Skálová, Margherita Gioria

**Affiliations:** 1 Department of Invasion Ecology, Institute of Botany of the Czech Academy of Sciences, Průhonice, Czech Republic; 2 Botany Unit, Department of Biology, University of Pisa, Pisa, Italy; 3 Department of Ecology, Faculty of Science, Charles University, Prague, Czech Republic

**Keywords:** Invasive species, Plant ecology

## Abstract

Soil seed viability and germinability dynamics can have a major influence on the establishment and spread of plants introduced beyond their native distribution range. Yet, we lack information on how temporal variability in these traits could affect the invasion process. To address this issue, we conducted an 8-year seed burial experiment examining seed viability and germinability dynamics for 21 invasive and 38 naturalized herbs in the Czech Republic. Seeds of most naturalized and invasive species persisted in the soil for several years. However, naturalized herbs exhibited greater seed longevity, on average, than invasive ones. Phylogenetic logistic models showed that seed viability (but not germinability) dynamics were significantly related to the invasion status of the study species. Seed viability declined earlier and more sharply in invasive species, and the probability of finding viable seeds of invasive species by the end of the experiment was low. Our findings suggest that invasive herbs might take advantage of high seed viability in the years immediately after dispersal, while naturalized species benefit from extended seed viability over time. These differences, however, are not sufficiently strong to explain the invasiveness of the species examined.

## Introduction

Invasions by alien plants introduced beyond their native distribution range by anthropogenic means represent a major component of global environmental change^1–3^. Knowledge of the mechanisms by which alien plants become established (naturalization) and spread from the original sources of introduction (invasions) is critical to predict new invasions and prevent potentially harmful introductions in an era of globalization and global environmental change^2,4^. Over the years, much attention has been paid to the search for species’ traits that may help predict the invasiveness of alien plants, i.e., the ability of a species to spread in the introduced geographic range after their successful establishment^5,6^. Reproductive traits have proved to play an especially important role in the naturalization and invasiveness of alien plants^7–9^.

Among reproductive traits, increasing evidence indicates that the formation of reservoirs of seeds that can persist in the soil over multiple regeneration seasons (persistent seed banks^10,11^) rather than those whose seeds remain viable for less than a year and not until the second germination season (transient seed banks), positively influences the probability of successful establishment of alien plants and contributes to the spread of naturalized populations^6,12,13^. In particular, recent analyses of global data from natural seed banks show that both the number of geographic regions where a species has become naturalized and the invasion status in some of these regions are better explained by the type (persistent vs. transient) and density of the soil seed bank than by other seed traits such as seed mass and dormancy^13^.

The mechanisms by which banking on persistent seeds can contribute to promoting naturalization and range expansion of alien species have been described by Gioria and colleagues^13,14^. The formation of persistent soil seed banks is one of the strategies that plants adopt to hedge against the risks of reproductive failure in unpredictable environments^15,16^ and persist in a community^17^. Persistent seed banks can mitigate the constraints to population regeneration associated with unfavourable or suboptimal conditions for germination and growth, such as intense competition, predation, or other biotic interactions, and with natural or anthropogenic disturbances^13,18–21^.

Seed banks act as reserves of seeds ready to germinate when suitable environmental conditions occur^14^. For alien plants, forming reserves of persistent seeds can be viewed as an important dimension of propagule pressure, that is the quantity, quality, and frequency of propagules (in this case, seeds) introduced in a system, and is a robust predictor of successful invasions^22,23^. Also, spreading mortality risks through time via persistent seed banks increases the probability of successful recruitment from seed by extending the windows of opportunity for successful germination, resulting in repeated episodes of establishment^13,24^. In grassland ecosystems and for many annual alien plants, these windows of opportunity for germination and seedling establishment often coincide with periods when competition for resources with native species is low^25,26^. This can facilitate establishment and range expansion in those alien species that are competitively inferior to natives^13,27^. The ability to form persistent seed banks is also important for alien species relying on short-distance dispersal and reproducing exclusively by seed^14^. For those species, dispersal through time might be more important than dispersal through space, given that they cannot escape negative biotic interactions or stochastic phenomena such as natural or anthropogenic disturbances^13^. In disturbed habitats, where alien plants are often successful^28–31^, persistent seed banks typically play a critical role in maintaining plant populations^21^.

As reserves of genetic variability^32,33^, persistent seed banks may contribute to determining the evolutionary response of plant populations to environmental unpredictability^16,34^, including climatic changes, although there is little evidence for this^35^. Rapid evolutionary changes towards improved seed survival in seed banks or optimization of the timing of germination could facilitate range expansions of alien plants^36^, especially when competition between alien and native species is strong^12,27^.

An effective way to evaluate whether seed persistence in the soil (or other species traits) could contribute to the invasiveness of alien plants is by comparing closely related invasive and naturalized (but non-invasive) species in their alien distribution range^5,37^. Studies of natural seed banks (versus those buried experimentally) generate useful information on the densities of seeds that a species can form under certain environmental conditions^13^. However, most of these studies classify seed banks only into transient versus persistent^21^. Moreover, this information is often estimated indirectly, based on the presence or absence of a species in the aboveground vegetation or is inferred from the depth at which viable seeds are found^10^. These two broad categories are important to predict whether a species can accumulate reserves of persistent seeds in the soil over time and make informed decisions on the need to develop control measures that target the seed bank as well as the standing vegetation of invasive plants^14,38^. However, understanding the role of the seed bank and regeneration from seeds in the persistence and expansion of invasive populations requires knowledge of how seed viability and germinability vary over time, which is best gained from controlled experiments.

In this respect, seed burial experiments conducted over multiple years can provide important insights into soil seed bank dynamics^39^ and their role in promoting the invasiveness of alien plants^40^. This information is especially critical for species that are known to form persistent seed banks but for which information on seed viability over time is uncertain^41,42^. Moreover, contrary to other approaches such as laboratory-controlled ageing^43,44^, seed burial experiments allow for seeds to be exposed to more ‘natural’ environmental and climatic conditions than in the laboratory, thus providing more realistic information on the loss of viability and germinability over time.

Presently, there is little information on how seed viability and germinability of alien plants change over time and how this might contribute to the spread of naturalized plants. To address this issue, we conducted an 8-year seed-burial experiment comparing seed viability and germinability dynamics of 21 herbaceous species that have been classified as invasive in the Czech Republic and 38 herbs that are considered naturalized but non-invasive^45^ (see Supplementary Table S1 for a list of these species and selected species traits), with such dynamics being modelled over a 10-year period, in a phylogenetic framework. We also evaluated the contribution of differences in life history (annual vs. perennial herbs) and seed mass in explaining patterns in seed viability and germinability over time since a correlation between these variables and seed persistence in the soil has been documented over large spatial scales^20,21,46,47^. Whether seed viability and germinability dynamics are related to information on the type of seed bank (transient vs. persistent) of the study species, collected under natural conditions, was also examined.

## Results

### Decline in seed viability and germinability in invasive and naturalized plants

**Figure 1:**
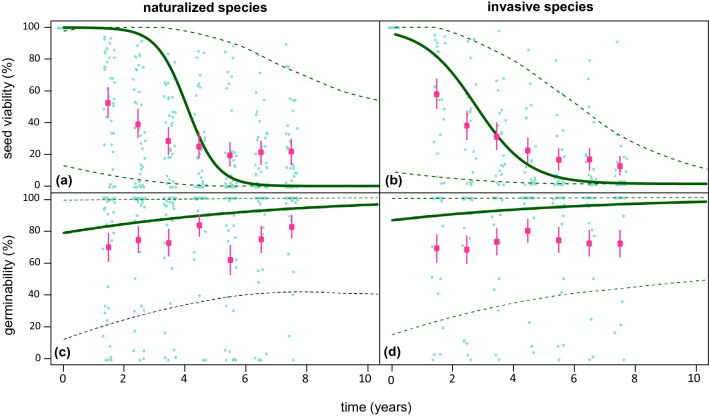
Observed mean seed viability and germinability (percentage) for (**a**,**c**) 38 naturalized and (**b**,**d**) 21 invasive species in each year of exhumation (small green dots) over 7.5 years from seed burial (time zero). Square pink symbols indicate observed mean seed viability values (± SE) for all species, within each group of species (naturalized vs. invasive). Overlapped are fitted values and credible intervals resulting from phylogenetically informed logistic models, modelling the decline in seed viability over 10 years.

**Figure 2:**
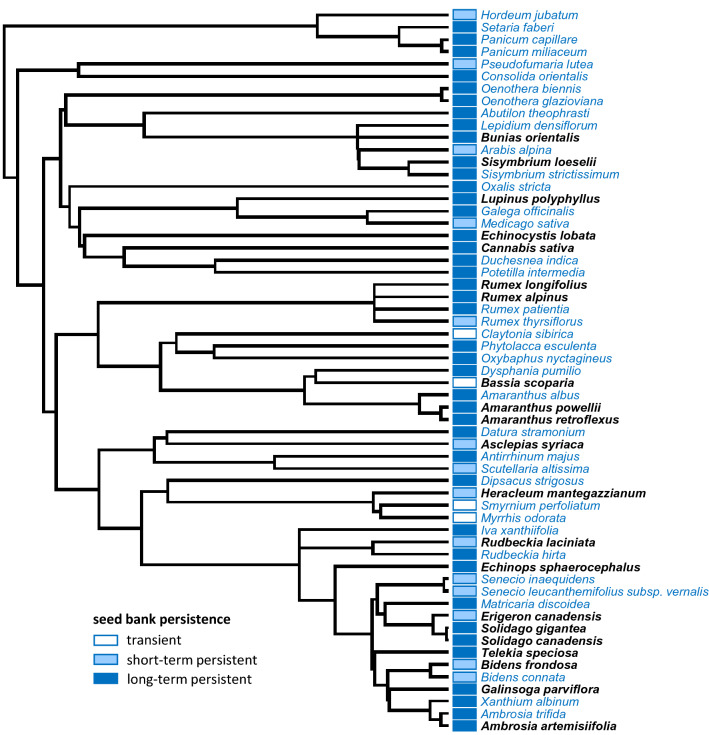
Plant phylogenetic tree and seed bank persistence derived from a 7.5-year seed burial experiment. Species in bold are classified as invasive in the Czech Republic, while those in blue are considered naturalized, according to the classification by Pyšek and colleagues (2012)^45^.

Mean pre-burial (fall 2012) seed viability did not differ significantly between invasive (89.3% ± 0.17) and naturalized alien species (85.8% ± 0.10) (see Supplementary Table S2). Seed viability decreased with increases in the time (years) since burial (Fig. 1). Seed viability was calculated as the percentage of viable seeds out of the total number of buried seeds in each of three replicate seed bags, for each year of exhumation (for a total of 21 seed bags per species). The observed average seed viability for each group of species (naturalized vs. invasive) was higher in invasive species up to 3.5 years since burial (Fig. 1). Phylogenetic logistic models revealed significantly different dynamics in seed viability for invasive and naturalized species (Fig. 1; Supplementary Table S3). These models were here used to model the decline in seed viability (and germinability) over 10 years while accounting for the shared evolutionary history among the 59 species used in the study (Fig. 2) and the effects of random factors related to the experimental design (Supplementary Table S3). Seed viability declined earlier and more sharply in invasive than naturalized plants and, eight years after burial, it was low for all invasive species but one (Fig. 1). Estimates of the time for seed viability to fall to 50% (*P*_50_) was 3.71 years (1.71–22.36) for invasive species versus 4.11 years (1.78–45.82) for naturalized species. Upper 95% credible intervals showed a very low probability of finding viable seeds after 8 years of burial in invasive species. On the contrary, this probability was moderate in naturalized species, with upper credible intervals embracing most naturalized species (Fig. 1; Supplementary Table S3). Seed germinability, which was here calculated as the percentage of seeds that germinated under laboratory conditions out of the number of viable seeds exhumed yearly (three replicate values per species), was, in contrast, similar in naturalized than invasive species throughout the duration of the experiment (Fig. 1), although, in naturalized species, most germinated seeds lie close to the upper 95% credible intervals (Supplementary Table S3).

### Effects of phylogeny and species traits

**Figure 3:**
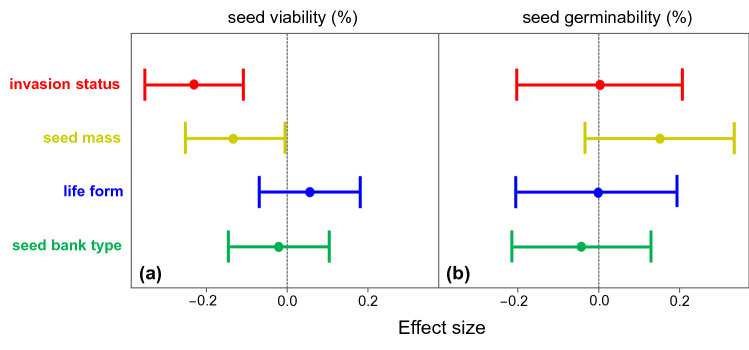
Effect of invasion status (invasive vs. naturalized), seed mass (log-transformed), life form (annual vs. perennial herb), and seed bank type (transient vs. persistent; GloSSBank^21^) on (**a**) seed viability and (**b**) germinability. The effects are presented as posterior means and 95% confidence intervals and were analysed as fixed effects in phylogenetic logistic mixed models. Dashed lines indicate zero effect.

In both logistic models of viability and germinability dynamics, the effect of phylogeny was strong (Supplementary Table S3), and a significant phylogenetic signal was observed in both traits, as indicated by values greater than 0.8 of the Pagel’s λ statistics (Supplementary Table S4). In relation to other species traits, phylogenetic logistic models showed a significant negative relationship between seed mass and seed viability as time increases, with the viability of large-seeded species declining more rapidly over time than that of small-seeded species (Supplementary Table S3). In contrast, the correlation between seed germinability and seed mass over time was not significant (Supplementary Table S3). Seeds of perennial herbaceous species also showed a greater decline in seed viability over time compared to those of annual herbs, although the difference was not significant (Supplementary Table S3). Models testing the effect of seed bank type (persistent vs. transient) collected under natural conditions and extracted from the Global Soil Seed Bank database (GloSSBank)^21^ showed similar seed viability and germinability percentages over time for species forming persistent or only transient seed banks (Supplementary Table S3). Size effects for invasiveness, seed mass, life form and seed bank type are presented in Fig. 3.

### Methodological considerations

Random effects associated with the experimental design (row number and sequence) had a minimal effect on seed viability and germinability dynamics (Supplementary Table S3), indicative of its suitability for addressing our research questions. Yet, we cannot exclude that some seeds in certain buried bags might have been lost through microenvironmental conditions that affected different replicates in different ways. Moreover, the conditions experienced by seeds in nylon bags differ from those encountered under natural conditions. This includes the lack of above-ground vegetation, inevitable disturbance that occurred when burying seeds in the soil, and the isolation of seeds in nylon mesh bags from soil biota^48^. These factors, however, have likely affected all species and replicates similarly. The wide variety of angiosperm lineages that we found in naturalized and invasive herbaceous species (Fig. 2) indicates that our results do not suffer from an a priori phylogenetic bias.

## Discussion

While global evidence shows that the ability to form persistent (rather than transient only) soil seed banks is an important species trait^21^ that increases the probability of alien species to become naturalized but also invasive beyond their native distribution range^13^, little is known about how temporal variability in the persistence of seeds could affect the invasion process. In an 8-year seed burial experiment, we found that a representative sample of invasive and naturalized herbaceous neophytes in the Czech Republic formed seed banks that persisted in the soil over several regeneration seasons, suggesting that long-lasting seed banks play an important role in the successful establishment of alien species, acting as sources of seeds ready to take advantage of environmental conditions to maximise their reproductive success. However, seed viability dynamics were significantly related to the invasion status of the study species (invasive vs. naturalized but non-invasive species).

Naturalized species exhibited greater seed longevity than invasive species, as revealed by higher percentages of seeds that were still viable towards the end of the experiment for most species. Moreover, seed viability declined earlier and more sharply in invasive than in naturalized species. Interestingly, seed viability was high only in one invasive species toward the end of experiment. These findings suggest that naturalized species might rely more on seed longevity for their persistence in a community compared to invasive species. Invasive species, on the contrary, might benefit from greater percentages of viable seeds in the years immediately after dispersal. This strategy would result in increased propagule pressure (in this case seeds) and could contribute positively to range expansion of alien populations due to a higher probability of encountering suitable conditions for germination and make them less dependent on maintaining naturalized populations through increased seed longevity. The observed differences in the decline in seed viability are clearly not sufficient to explain the status of the species classified as invasive in our study, which is likely dependent on other factors, such as the dispersal characteristics of the study species and external factors^8,12^. Moreover, long-distance dispersal of invasive species is often human-mediated, thus affecting the relative importance of species traits (or variation in species traits) in the invasion process^8,12,13^. Yet, for some of these species, seed viability dynamics could have provided them with some temporal advantages over other alien or native species that facilitated their establishment and spread.

Seed germinability, defined here as the fraction of viable seeds germinating yearly after seed exhumation, did not differ significantly between naturalized and invasive species and was generally high (> 60%) in both groups of species over the duration of the experiment. This further supports evidence of the importance of banking on persistent seeds for both naturalized and invasive plants as a strategy to hedge against unpredictable environmental conditions in the introduced range^12,13^ as well as a capacity of both groups of species to adjust the timing of germination until the environment is perceived as ‘safe’^18^. However, germination of high fractions of viable seeds may increase the vulnerability of a species to unpredictable post-germination conditions^13,15,16,49^, especially when it occurs early in the growing season^25,26,50,51^. At such times, in fact, the risk that conditions that promoted germination might not reflect the actual beginning of the germination season is high^26^. While risky, this strategy often allows alien species to germinate and take up resources earlier than native species, thus avoiding or mitigating any potential negative effect of competitive interactions with native species^26^ and potentially promoting invasiveness.

A tendency for higher seed viability and germinability observed in invasive species in the first few years after seed burial compared to naturalized species suggests that producing many viable, well-germinating seeds in the regeneration seasons following dispersal is a strategy that could contribute to promote the spread of naturalized populations by increasing the probability of successful recruitment from the seed bank. This is in line with evidence that producing high numbers of viable seeds represents an important factor in the invasion process^9,52^, especially small seeds that require light for germination and that can germinate rapidly soon after disturbance^46,53–57^. Moreover, germination of large numbers of seeds of alien plants might prevent the germination of the seed of native plants, especially when resulting in the formation of dense mats of seedlings germinating earlier than seeds of native plants^42,58^, and/or when they outcompete seedlings of native plants via a more effective uptake of resources^14,27^.

Phylogenetic relatedness strongly influenced seed viability and germinability dynamics, confirming the importance of evolutionary history as a determinant of the germination strategies^59–61^ and seed-banking strategies (persistence vs. transience) of seed plants^21^. This was confirmed by a significant phylogenetic signal observed both for seed viability and germinability, indicative that these properties are not randomly distributed across the phylogeny.

As for the effects of other species traits, similar seed viability dynamics were observed for annual and perennial alien herbs. Long-term evidence shows that persistent seed banks serve as a bet-hedging strategy for short-lived species, which depend heavily on the ability to disperse through time for survival^15,16,20^. Seed persistence is generally regarded as higher in annual than perennial herbs^20^ and evidence, but in a phylogenetic framework, shows that annual herbs are more likely to form persistent seed banks than perennial herbs^21^. Yet, our findings are consistent with global-scale evidence that the ability to form persistent seed bank is important not only for short-lived herbs but also for but also for perennial herbs^13^. We also found a weak correlation between viability and germinability dynamics on the one hand and the ability of a species to form transient versus persistent seed banks under natural conditions. This likely reflects the fact that discrete traits such as transience versus persistence do not encompass information on how seed persistence varies over time.

We found a negative correlation between seed mass and seed viability over time, consistent with long-term evidence that large seeds persist less in the soil than small seeds^21,46,62^. In general, however, it has been shown that seed mass is not a robust predictor of the ability of plants to disperse in time^21,47,63,64^. Moreover, seed mass has been reported to contribute to naturalization and invasiveness in opposing ways, with greater naturalization success in large-seeded species and greater invasiveness in small-seeded species^22,65^. Also, the contribution of seed mass to invasiveness typically varies with the spatial scale of the study and among life forms (herbs vs. woody)^22,23,37^. Global-scale phylogenetic analyses have recently revealed a weak effect of seed mass on the naturalization and invasion status of a species relatively to that of seed bank persistence^13^. Besides its potential effects on the dispersal capacity of a species^8^, it is possible that the effect of seed mass on naturalization or invasiveness might be associated with a slower decline in seed viability for small- rather than large-seeded species, as we found in our study.

To conclude, our findings suggest that invasive alien herbs take advantage of higher seed viability in the first few seasons following dispersal while naturalized species maintain naturalized populations through extended seed viability and germinability over time. While differences in viability dynamics of seeds in the soil could contribute to promoting invasiveness at the initial stages of the invasion process, they do not provide a robust explanation for the successful spread of naturalized plants. Our findings also point to the need for a better understanding of how reproductive traits vary over time and how temporal variability affects naturalization and invasiveness; on the contrary, the use of discrete species traits summarizing the ability (or the lack of it) of a species to persist in the soil as viable seeds for multiple regeneration seasons might fail to capture how temporal variability in seed persistence might impact on the ability of alien species to progress along the invasion process.

From a broader perspective, this study generates new information on seed longevity and the seed-banking strategies of many alien species that have become a common feature of central-European plant communities. For instance, the highly invasive *Ambrosia artemisiifolia* showed the highest seed viability (90%) and germinability (100%) recorded at the end of the experiment, providing evidence that the seed stage might play a critical role in determining the invasiveness of this species. Species-level information on seed persistence in the soil over time is key to estimating the potential reserve of viable seeds that these species may accumulate over time and develop effective management measures. From a practical point of view, our results indicate that managing naturalized or invasive populations of most alien herbs requires the development of control measures that need to be applied over several years, with the aim of exhausting the seed bank and preventing regeneration from seed after removal of the invaders from the vegetation. While naturalized herbs showed different seed viability dynamics than invasive herbs, it is possible that these species might become invasive in the future, given the highly dynamic nature of the invasion process, as revealed by changes in the status of the Czech alien flora^45,66^. It is likewise possible, however, that the advantage provided by high seed viability and germinability is only transient and that the distribution of invasive species that rapidly lose seed viability in the soil declines over time, especially in the presence of natural or anthropogenic disturbances that alter direct and indirect interactions between the invasive and native species. Understanding relationships between seed viability (and germinability) dynamics and the long-term persistence of invasive populations is a topic worthy of investigation.

Ultimately, the ability of alien species to form persistent banks of viable seeds will likely play a greater role in the success of alien plants in the future. Increasing evidence shows that persistent seed banks mediate the response of plants to environmental changes^34^ and promote range shifts under a changing climate^35^. Climatic changes could be especially beneficial to those species capable of exploiting conditions created by extensions or shifts in the growing/germination season(s), such as those possessing seeds with broad germination requirements and/or those characterized by asynchronous seed germination^25^. This makes understanding how the persistence of alien seeds change over time under local and global environmental changes even more imperative.

## Methods

### Study species

To assess whether invasive and naturalized alien herbs differ in seed viability and germinability over time, we selected 59 neophyte species (introduced after the year 1500^66,67^) that differ in their invasion status in the Czech Republic; of those, 21 are classified as invasive and 38 as naturalized but non-invasive^45^. For each species, we included information on its lifecycle (annual vs. perennial herbs^45^), seed mass, and seed bank type. Seed mass (g) was measured by weighing four sets of 25 propagules from each locality and calculating the average weight of one set^52^. Information on seed bank type (transient vs. persistent) was extracted from the GloSSBank database, a global database of soil seed bank data^21^. A full species list with information on the invasion status of each species (invasive vs. naturalized) and selected species traits is provided in Supplementary Table S1. The taxonomic status of each species was validated using the World Flora Online database^68^ (http://www.worldfloraonline.org).

### Seed collection and pre-burial seed viability

Seeds were collected from naturalized or invasive populations from July to October 2012, depending on the suitable seed collection time for each species (see Supplementary Table S5 for a list of localities, habitats, and dates of the collection). Since the 59 species are common in the Czech Republic, their identity was established by members of the team and voucher specimens were not collected. In total, seeds were collected from 126 populations. Most of these populations were located on private land and permission to collect seeds had been previously obtained from private landowners. Seeds of invasive *Rumex* species and naturalized *Matricaria discoidea* and *Myrrhis odorata* were collected from Krkonoše National Park, and permission to collect seeds of these species, which are not protected by law, had been granted from the relevant authority. After collection, seeds were dry stored in paper bags at room temperature until the beginning of the burial experiment. Pre-burial seed viability was tested using four replicates of 25 seeds for each species, which were placed in 6-cm Petri dishes on a double layer of Whatman no. 2 filter paper. Seeds were incubated in growth chambers at alternating 25°/10 °C (12 h:12 h photoperiod) and an average irradiance of 0,37 photons. Seeds of species known to possess physiologically dormant seeds were cold stratified at 3 − 5 °C in darkness for three months prior to germination tests. Seed germination was counted three times a week for three weeks. Seed germinating over this three-week period were classified as germinated. Non-germinated seeds were first stimulated by gibberellic acid (1%) and the remaining non-germinated seeds were tested for viability by staining the dissected seed with tetrazolium (1% 2,3,5-triphenyltetrazolium chloride); these seeds were classified as viable. Information on pre-burial seed viability, composed of germinated and non-germinated but viable seeds, provided information on whether invasive and naturalized species differ in seed viability before seed burial.

### Burial experiment

Seeds were buried at the common garden facility of the Institute of Botany, Průhonice, Czech Republic (49°59′40''N; 14°3′57''E; alt. 330 m a.s.l.), in compliance with institutional regulations and national legislation on invasive species (Act No. 114/1992 Sb. on nature and landscape protection). All seeds were destroyed after exhumation and germination tests. Mixed soil samples were collected from three positions within the burial experimental field (mowed lawn) at a depth of 7–10 cm (same depth at which seeds were buried). Average soil properties in these samples were: dry matter: 98.13%; pH [KCl]: 7.52; total N: 0.11%, total C: 1.07%, Ca 3316.39, Mg: 85.72, K: 78.17, P: 43.0 mg/kg. In November 2012, we buried three seed bags for each species (replicates) containing 100 seeds each (with few exceptions of plants poor in seed), in each year of planned exhumation (7 years), for a total of 21 seed bags for each species (Supplementary Table S2). Each bag contained a mixture of seeds from one to three populations. Only visually undamaged seeds were used in the experiment. Individual seed bags were buried according to a random design that did not place any of the three replicates nearby. To prevent seed predation, each seed sample was placed into nylon bags (6 cm × 8 cm), with a rectangular mesh of 266 × 162 μm. Bags were buried 7 to 10 cm below the soil surface, in stripes of six bags. Soil temperature (on the soil surface, 5 cm above, and 5 cm below) and soil moisture were monitored daily using a datalogger throughout the experiment (Supplementary Fig. S1).

### Seed exhumation

Seeds buried in November 2012 were exhumed each spring (from the end of March to the beginning of April) for 7 years, from 2014 to 2020. After exhumation, seeds were tested for viability in germination experiments using the same procedure used to evaluate pre-burial viability. The content of each bag was spread out in a Petri dish, and good (firm) seeds and decayed seeds were counted under a stereomicroscope. Firm seeds were incubated at alternating 25°/10 °C (12 h:12 h photoperiod) for 3 weeks. After 3 weeks, germination of non-germinated seeds was stimulated by gibberellic acid, and seeds not yet germinated were stained with tetrazolium, using the procedure described for the pre-burial experiment. Small seeds of *Erigeron canadensis*, *Duchesnea indica*, *Dysphania pumilio*, *Potentilla intermedia*, *Sisymbrium* and *Solidago* species were tested for viability by squeezing with a tweezer and not by staining with tetrazolium. Firm seeds were supposed to be viable. Differences between the number of buried seeds and that of exhumed seeds in each bag could be due to the loss of seeds through germination, decay, or natural enemies. These seeds were collectively classified as dead at the date of exhumation since it was not possible to differentiate among these processes. For each year, exhumed seeds of each species/replicate were classified into three categories: germinated, viable (but non-germinated), and dead. From these data, we derived three variables for each sample: *seed viability*, the percentage of viable seeds of the total number of buried seeds; *seed germinability*, the percentage of seeds that germinated under laboratory conditions among the number of viable seeds exhumed; and *seed germination*, the percentage of seeds that germinated naturally under laboratory conditions without the stimulation with gibberellic acid, of the total number of buried seeds. At the end of the burial experiment, the seed bank of each species (*seed bank persistence*) was classified as transient, short-term persistent, and long-term persistent (sensu Thompson and colleagues^10^). Transient seed banks were those for which no viable seeds were found after 1.5 years since burial, while long-term persistent seed banks were those for which viable seeds were still recorded after 5.5 years since burial.

### Data analyses

The decline in seed viability and germinability over time in invasive and naturalized species was modelled using logistic generalized mixed models with Bayesian estimation (Markov Chain Monte Carlo generalized linear mixed models, MCMCglmms^69^). Specifically, seed viability and germinability were modelled, separately, as functions of the invasion status of the study species (invasive vs. naturalized but non-invasive). The effects of seed mass [log-transformed], life form (annual vs. perennial herbs), and seed-bank type (transient vs. persistent) on seed viability and germinability were also tested, in separate logistic MCMCglmm models, given the importance of these traits in determining seed persistence in the seed bank^21^. In models of seed viability, we estimated *P*_50_ (the time taken for seed viability to fall to 50%) and fitted seed survival curves to the raw data using the viability equation proposed by Ellis and Roberts^70^, with *ν* = *K*_i_–*p*/*σ*, where *ν* is the viability of each of three seed samples (bags) buried after *p* years of burial, *K*_i_ is seed viability at time zero (pre-burial viability), and *σ* is the standard deviation of the normal distribution of non-viable seeds in time (years)^70,71^. An estimate of the time taken for seed viability to fall to 50% (*P*_50_) was calculated by solving for *p* when *v* = 50. While this formula has been traditionally used to estimate seed longevity of seeds of individual species stored under international conservation standards^70^, here we used it to compare the decline in viability for naturalized and invasive species and not to provide seed longevity parameters to be used in future comparative studies. In these models we assumed that all seeds were viable at the time of burial since viability and germinability were often higher than the estimated pre-burial seed viability. This is a solid assumption, given that seeds were visually inspected prior to seed burial.

Since seed persistence (versus transience) in the soil is phylogenetically structured in angiosperms^21^, we accounted for shared evolutionary history by including the reconstructed phylogeny among the random effects. The phylogenetic tree was constructed using the R package ‘V. PhyloMaker’^72,73^, using the *bind.relative* function to attach taxa absent from the implemented mega-tree by Smith and Brown^74^ to their designated genus. Species identity, triplets (three replicates for each seed exhumation year), row sequence, and row number, indicative of the coordinates of each triplet for each species in the burial experiment, were also included among the random effects.

We used weakly informative priors in all models, fixing the residual covariance matrix for binary traits while using parameter expanded priors for the random effects for continuous response variables. Each model was run for 1,000,000 MCMC steps, with an initial burn-in phase of 10,000 and a thinning interval of 100^75^, resulting, on average, in 9000 posterior distributions. From the resulting posterior distributions, we calculated the posterior mean, posterior mode, and lambda, and 95% Credible Intervals (CI). Values of lambda were interpreted as how variation in the fixed variables are correlated with phylogenetic relatedness in the study species^76,77^. The significance of model parameters was estimated by examining CIs; parameters with CIs overlapping with zero were considered not significant. The above models were then used to predict seed viability and seed germinability over a 0 to 10 years period using the function *predict* in the MCMCglmm package^69^. All analyses were conducted in the R software environment (v. 4.1.1^78^).

## Supplementary Information

Supplementary Information.

## Data Availability

All data generated or analysed during this study are included in this published article [and its supplementary information files].
