# Supplementary material for: Long-term seed burial reveals differences in the seed-banking strategies of naturalized and invasive alien herbs
Source: Sci Rep. 2022 May 25;12:8859. doi: 10.1038/s41598-022-12884-0 (PMC9132925; doi:10.1038/s41598-022-12884-0)
Supplement: Supplementary file 1 — Supplementary Information. [file 41598_2022_12884_MOESM1_ESM.docx]

L. Moravcová, A. Carta, P. Pyšek, H. Skálová & M. Gioria M. Long-term seed burial reveals differences in the seed-banking strategies of naturalized and invasive alien herbs.

**Supplementary Information**

**Supplementary Table S1.** List of 59 species used in our seed burial experiment, together with their invasive status in the Czech Republic (from Pyšek et al. 2012), life form (annual versus perennial herbs), seed bank type extracted from the GloSSBank database (Gioria et al. 2020), and seed mass (, measured at the beginning of the experiment.

| **species** | **family** | **invasive status** | **life form (herbs)** | **seed bank type (GloSSBank)** | **25 seed mass**  **(g)** |
| --- | --- | --- | --- | --- | --- |
| *Abutilon theophrasti* | Malvaceae | naturalized | annual | transient | 0.2462 |
| *Amaranthus albus* | Amaranthaceae | naturalized | annual | persistent | 0.008 |
| *Amaranthus powellii* | Amaranthaceae | invasive | annual | - | 0.0133 |
| *Amaranthus retroflexus* | Amaranthaceae | invasive | annual | persistent | 0.0112 |
| *Ambrosia artemisiifolia* | Compositae | invasive | annual | persistent | 0.1218 |
| *Ambrosia trifida* | Compositae | naturalized | annual | persistent | 0.3993 |
| *Antirrhinum majus* | Plantaginaceae | naturalized | perennial | transient | 0.0032 |
| *Arabis alpina* | Brassicaceae | naturalized | perennial | transient | 0.0075 |
| *Asclepias syriaca* | Apocynaceae | invasive | perennial | - | 0.2562 |
| *Bassia scoparia* | Amaranthaceae | invasive | annual | persistent | 0.0305 |
| *Bidens connata* | Compositae | naturalized | annual | persistent | 0.1055 |
| *Bidens frondosa* | Compositae | invasive | annual | persistent | 0.0537 |
| *Bunias orientalis* | Brassicaceae | invasive | perennial | - | 0.8179 |
| *Cannabis sativa* | Cannabaceae | invasive | annual | - | 0.2321 |
| *Claytonia sibirica* | Montiaceae | naturalized | annual | transient | 0.0204 |
| *Consolida orientalis* | Ranunculaceae | naturalized | annual | - | 0.0487 |
| *Datura stramonium* | Solanaceae | naturalized | annual | persistent | 0.1907 |
| *Dipsacus strigosus* | Caprifoliaceae | naturalized | perennial | - | 0.1499 |
| *Duchesnea indica* | Rosaceae | naturalized | perennial | persistent | 0.007 |
| *Dysphania pumilio* | Amaranthaceae | naturalized | annual | - | 0.0022 |
| *Echinocystis lobata* | Cucurbitaceae | invasive | annual | - | 6.179 |
| *Echinops sphaerocephalus* | Compositae | invasive | perennial | - | 0.5973 |
| *Erigeron canadensis* | Compositae | invasive | annual | persistent | 0.0009 |
| *Galega officinalis* | Fabaceae | naturalized | perennial | - | 0.164 |
| *Galinsoga parviflora* | Compositae | invasive | annual | persistent | 0.0047 |
| *Heracleum mantegazzianum* | Apiaceae | invasive | perennial | persistent | 0.3795 |
| *Hordeum jubatum* | Poaceae | naturalized | annual | persistent | 0.059 |
| *Iva xanthiifolia* | Compositae | naturalized | annual | - | 0.0242 |
| *Lepidium densiflorum* | Brassicaceae | naturalized | annual | transient | 0.0075 |
| *Lupinus polyphyllus* | Fabaceae | invasive | perennial | - | 0.5649 |
| *Matricaria discoidea* | Compositae | naturalized | annual | persistent | 0.0033 |
| *Medicago sativa* | Fabaceae | naturalized | perennial | persistent | 0.323 |
| *Myrrhis odorata* | Apiaceae | naturalized | perennial | - | 0.9669 |
| *Oenothera biennis* | Onagraceae | naturalized | perennial | persistent | 0.016 |
| *Oenothera glazioviana* | Onagraceae | naturalized | perennial | - | 0.007 |
| *Oxalis stricta* | Oxalidaceae | naturalized | perennial | persistent | 0.0048 |
| *Oxybaphus nyctagineus* | Nyctaginaceae | naturalized | perennial | - | 0.1553 |
| *Panicum capillare* | Poaceae | naturalized | annual | persistent | 0.0138 |
| *Panicum miliaceum* | Poaceae | naturalized | annual | transient | 5 |
| *Phytolacca esculenta* | Phytolaccaceae | naturalized | perennial | - | 0.2698 |
| *Potentilla intermedia* | Rosaceae | naturalized | perennial | - | 0.0032 |
| *Pseudofumaria lutea* | Papaveraceae | naturalized | perennial | - | 0.0359 |
| *Rudbeckia hirta* | Compositae | naturalized | perennial | persistent | 0.0131 |
| *Rudbeckia laciniata* | Compositae | invasive | perennial | - | 0.0726 |
| *Rumex alpinus* | Polygonaceae | invasive | perennial | - | 0.0536 |
| *Rumex longifolius* | Polygonaceae | invasive | perennial | persistent | 0.061 |
| *Rumex patientia* | Polygonaceae | naturalized | perennial | - | 0.116 |
| *Rumex thyrsiflorus* | Polygonaceae | naturalized | perennial | - | 0.0198 |
| *Scutellaria altissima* | Lamiaceae | naturalized | perennial | - | 0.0355 |
| *Senecio inaequidens* | Compositae | naturalized | perennial | - | 0.0055 |
| *Senecio leucanthemifolius* subsp. *vernalis* | Compositae | naturalized | annual | - | 0.0055 |
| *Setaria faberi* | Poaceae | naturalized | annual | transient | 0.0464 |
| *Sisymbrium loeselii* | Brassicaceae | invasive | annual | - | 0.0022 |
| *Sisymbrium strictissimum* | Brassicaceae | naturalized | perennial | - | 0.0101 |
| *Smyrnium perfoliatum* | Apiaceae | naturalized | perennial | - | 0.2105 |
| *Solidago canadensis* | Compositae | invasive | perennial | transient | 0.0018 |
| *Solidago gigantea* | Compositae | invasive | perennial | persistent | 0.0046 |
| *Telekia speciosa* | Compositae | invasive | perennial | - | 0.014 |
| *Xanthium albinum* | Compositae | naturalized | annual | - | 8.6231 |

References

Gioria, M., Pyšek, P., Baskin, C., & Carta, A. Phylogenetic relatedness mediates persistence and density of soil seed banks. *J Ecol.* **108**, 2121–2131 (2020).

Pyšek, P. et al. Catalogue of alien plants of the Czech Republic (2nd edition): checklist update, taxonomic diversity and invasion patterns. *Preslia* **84**, 155–255 (2012).

**Supplementary Table S2.** Mean seed viability percentage (percentage of viable seeds of the total number of buried seeds), mean seed germinability (percentage of germinated seeds of the number of viable seeds), and mean germination (percentage of germinated seeds of the total number of buried seeds), for each year of exhumation. Seeds were buried in 2012. Based on these findings, the seed bank of each species was classified as long-term persistent, short-term persistent, and transient (sensu Thompson et al. 1997). The number of buried seeds for each species in each of three sample bags and pre-burial seed viability are also reported.

| **species** | **no. buried seeds per sample bag** | **pre-burial viability (%)** | **years of burial** | **mean seed viability (% of buried seeds)** | **mean seed germinability (% of viable seeds)** | **mean seed germination (% of buried seeds)** | **seed bank persistence** |
| --- | --- | --- | --- | --- | --- | --- | --- |
| *Abutilon theophrasti* | 100 | 98 | 1.5 | 91.67 | 93.28 | 85.33 | long-term persistent |
|  | 100 |  | 2.5 | 80.67 | 97.45 | 78.67 |  |
|  | 100 |  | 3.5 | 64.33 | 63.02 | 41.00 |  |
|  | 100 |  | 4.5 | 79.67 | 83.32 | 66.33 |  |
|  | 100 |  | 5.5 | 79.33 | 73.35 | 60.00 |  |
|  | 100 |  | 6.5 | 70.33 | 77.56 | 54.33 |  |
|  | 100 |  | 7.5 | 54.33 | 86.56 | 46.67 |  |
| *Amaranthus albus* | 100 | 96 | 1.5 | 40.00 | 98.61 | 39.67 | long-term persistent |
|  | 100 |  | 2.5 | 35.00 | 100.00 | 35.00 |  |
|  | 100 |  | 3.5 | 39.00 | 100.00 | 39.00 |  |
|  | 100 |  | 4.5 | 2.00 | 66.67 | 2.00 |  |
|  | 100 |  | 5.5 | 5.00 | 57.14 | 3.00 |  |
|  | 100 |  | 6.5 | 32.67 | 61.11 | 32.33 |  |
|  | 100 |  | 7.5 | 32.67 | 33.33 | 32.67 |  |
| *Amaranthus powellii* | 100 | 99 | 1.5 | 81.00 | 98.12 | 79.67 | long-term persistent |
|  | 100 |  | 2.5 | 63.33 | 90.16 | 59.00 |  |
|  | 100 |  | 3.5 | 12.33 | 89.49 | 10.67 |  |
|  | 100 |  | 4.5 | 14.67 | 64.23 | 13.67 |  |
|  | 100 |  | 5.5 | 2.00 | 33.33 | 2.00 |  |
|  | 100 |  | 6.5 | 5.00 | 53.33 | 4.33 |  |
|  | 100 |  | 7.5 | 9.33 | 66.67 | 9.33 |  |
| *Amaranthus retroflexus* | 100 | 95 | 1.5 | 80.33 | 70.01 | 61.33 | long-term persistent |
|  | 100 |  | 2.5 | 53.00 | 10.01 | 5.67 |  |
|  | 100 |  | 3.5 | 39.00 | 88.15 | 33.33 |  |
|  | 100 |  | 4.5 | 7.67 | 33.33 | 7.67 |  |
|  | 100 |  | 5.5 | 0.33 | 33.33 | 0.33 |  |
|  | 100 |  | 6.5 | 0.67 | 33.33 | 0.33 |  |
|  | 100 |  | 7.5 | 5.33 | 66.67 | 5.33 |  |
| *Ambrosia artemisiifolia* | 100 | 99 | 1.5 | 94.33 | 100.00 | 94.33 | long-term persistent |
|  | 100 |  | 2.5 | 91.67 | 98.50 | 90.33 |  |
|  | 100 |  | 3.5 | 97.00 | 100.00 | 97.00 |  |
|  | 100 |  | 4.5 | 85.00 | 100.00 | 85.00 |  |
|  | 100 |  | 5.5 | 78.33 | 100.00 | 78.33 |  |
|  | 100 |  | 6.5 | 78.33 | 100.00 | 78.33 |  |
|  | 100 |  | 7.5 | 90.00 | 100.00 | 90.00 |  |
| *Ambrosia trifida* | 100 | 48 | 1.5 | 44.67 | 100.00 | 44.67 | long-term persistent |
|  | 100 |  | 2.5 | 44.33 | 97.62 | 43.33 |  |
|  | 100 |  | 3.5 | 25.33 | 96.62 | 24.67 |  |
|  | 100 |  | 4.5 | 35.00 | 100.00 | 35.00 |  |
|  | 100 |  | 5.5 | 36.33 | 86.94 | 29.67 |  |
|  | 100 |  | 6.5 | 15.00 | 100.00 | 15.00 |  |
|  | 100 |  | 7.5 | 23.00 | 100.00 | 23.00 |  |
| *Antirrhinum majus* | 100 | 62 | 1.5 | 34.33 | 53.86 | 26.67 | long-term persistent |
|  | 100 |  | 2.5 | 20.00 | 32.78 | 19.67 |  |
|  | 100 |  | 3.5 | 3.00 | 33.33 | 3.00 |  |
|  | 100 |  | 4.5 | 2.33 | 100.00 | 2.33 |  |
|  | 100 |  | 5.5 | 1.67 | 33.33 | 0.33 |  |
|  | 100 |  | 6.5 | 9.33 | 32.14 | 9.00 |  |
|  | 100 |  | 7.5 | 1.67 | 66.67 | 1.67 |  |
| *Arabis alpina* | 100 | 94 | 1.5 | 25.00 | 1.08 | 0.33 | short-term persistent |
|  | 100 |  | 2.5 | 12.00 | 1.85 | 0.67 |  |
|  | 100 |  | 3.5 | 6.67 | 11.67 | 2.33 |  |
|  | 100 |  | 4.5 | 0.33 | 33.33 | 0.33 |  |
|  | 100 |  | 5.5 | 0.00 | 0.00 | 0.00 |  |
|  | 100 |  | 6.5 | 1.67 | 0.00 | 0.00 |  |
|  | 100 |  | 7.5 | 0.00 | 0.00 | 0.00 |  |
| *Asclepias syriaca* | 100 | 94 | 1.5 | 84.67 | 100.00 | 84.67 | short-term persistent |
|  | 100 |  | 2.5 | 32.33 | 99.12 | 32.00 |  |
|  | 100 |  | 3.5 | 31.33 | 100.00 | 31.33 |  |
|  | 100 |  | 4.5 | 23.00 | 100.00 | 23.00 |  |
|  | 100 |  | 5.5 | 1.33 | 33.33 | 1.00 |  |
|  | 100 |  | 6.5 | 0.00 | 0.00 | 0.00 |  |
|  | 100 |  | 7.5 | 0.00 | 0.00 | 0.00 |  |

**Supplementary Table S2.** Continued

| **species** | **no. buried seeds per sample bag** | **pre-burial viability (%)** | **years of burial** | **mean seed viability (% of buried seeds)** | **mean seed germinability (% of viable seeds)** | **mean seed germination (% of buried seeds)** | **seed bank persistence** |
| --- | --- | --- | --- | --- | --- | --- | --- |
| *Bassia scoparia* | 100 | 89 | 1.5 | 0.67 | 33.33 | 0.67 | transient |
|  | 100 |  | 2.5 | 0.00 | 0.00 | 0.00 |  |
|  | 100 |  | 3.5 | 0.00 | 0.00 | 0.00 |  |
|  | 100 |  | 4.5 | 0.00 | 0.00 | 0.00 |  |
|  | 100 |  | 5.5 | 0.00 | 0.00 | 0.00 |  |
|  | 100 |  | 6.5 | 0.00 | 0.00 | 0.00 |  |
|  | 100 |  | 7.5 | 0.00 | 0.00 | 0.00 |  |
| *Bidens connata* | 100 | 90 | 1.5 | 85.00 | 100.00 | 85.00 | short-term persistent |
|  | 100 |  | 2.5 | 3.00 | 66.67 | 3.00 |  |
|  | 100 |  | 3.5 | 2.00 | 33.33 | 2.00 |  |
|  | 100 |  | 4.5 | 1.00 | 66.67 | 1.00 |  |
|  | 100 |  | 5.5 | 0.33 | 0.00 | 0.00 |  |
|  | 100 |  | 6.5 | 0.00 | 0.00 | 0.00 |  |
|  | 100 |  | 7.5 | 0.00 | 0.00 | 0.00 |  |
| *Bidens frondosa* | 100 | 99 | 1.5 | 87.00 | 100.00 | 87.00 | short-term persistent |
|  | 100 |  | 2.5 | 40.33 | 99.09 | 39.67 |  |
|  | 100 |  | 3.5 | 9.00 | 100.00 | 9.00 |  |
|  | 100 |  | 4.5 | 2.00 | 33.33 | 2.00 |  |
|  | 100 |  | 5.5 | 3.67 | 95.83 | 3.33 |  |
|  | 100 |  | 6.5 | 0.33 | 33.33 | 0.33 |  |
|  | 100 |  | 7.5 | 0.00 | 0.00 | 0.00 |  |
| *Bunias orientalis* | 147 | 100 | 1.5 | 94.33 | 3.09 | 2.95 | long-term persistent |
|  | 147 |  | 2.5 | 80.73 | 11.99 | 10.66 |  |
|  | 147 |  | 3.5 | 66.21 | 2.94 | 2.04 |  |
|  | 147 |  | 4.5 | 48.53 | 22.98 | 10.88 |  |
|  | 147 |  | 5.5 | 34.69 | 8.97 | 4.54 |  |
|  | 147 |  | 6.5 | 39.91 | 1.78 | 0.91 |  |
|  | 147 |  | 7.5 | 31.75 | 21.43 | 6.80 |  |
| *Cannabis sativa* | 100 | 98 | 1.5 | 53.00 | 40.87 | 24.33 | long-term persistent |
|  | 100 |  | 2.5 | 47.67 | 70.57 | 33.67 |  |
|  | 100 |  | 3.5 | 37.00 | 43.29 | 18.33 |  |
|  | 100 |  | 4.5 | 41.67 | 100.00 | 41.67 |  |
|  | 100 |  | 5.5 | 43.33 | 41.59 | 19.00 |  |
|  | 100 |  | 6.5 | 48.67 | 84.43 | 41.00 |  |
|  | 100 |  | 7.5 | 17.33 | 36.06 | 8.00 |  |
| *Claytonia sibirica* | 100 | 93 | 1.5 | 0.00 | 0.00 | 0.00 | transient |
|  | 100 |  | 2.5 | 0.00 | 0.00 | 0.00 |  |
|  | 100 |  | 3.5 | 0.00 | 0.00 | 0.00 |  |
|  | 100 |  | 4.5 | 0.00 | 0.00 | 0.00 |  |
|  | 100 |  | 5.5 | 0.00 | 0.00 | 0.00 |  |
|  | 100 |  | 6.5 | 0.00 | 0.00 | 0.00 |  |
|  | 100 |  | 7.5 | 0.00 | 0.00 | 0.00 |  |
| *Consolida orientalis* | 100 | 99 | 1.5 | 83.33 | 0.00 | 0.00 | long-term persistent |
|  | 100 |  | 2.5 | 80.67 | 0.00 | 0.00 |  |
|  | 100 |  | 3.5 | 82.67 | 0.00 | 0.00 |  |
|  | 100 |  | 4.5 | 84.33 | 0.00 | 0.00 |  |
|  | 100 |  | 5.5 | 84.00 | 0.00 | 0.00 |  |
|  | 100 |  | 6.5 | 82.33 | 0.00 | 0.00 |  |
|  | 100 |  | 7.5 | 75.33 | 0.00 | 0.00 |  |
| *Erigeron canadensis* | 100 | 88 | 1.5 | 4.67 | 33.33 | 4.33 | short-term persistent |
|  | 100 |  | 2.5 | 7.00 | 33.33 | 7.00 |  |
|  | 100 |  | 3.5 | 10.33 | 48.89 | 5.00 |  |
|  | 100 |  | 4.5 | 2.67 | 66.67 | 2.67 |  |
|  | 100 |  | 5.5 | 3.67 | 88.89 | 3.33 |  |
|  | 100 |  | 6.5 | 0.00 | 0.00 | 0.00 |  |
|  | 100 |  | 7.5 | 0.00 | 0.00 | 0.00 |  |
| *Datura stramonium* | 100 | 98 | 1.5 | 26.00 | 30.22 | 6.67 | long-term persistent |
|  | 100 |  | 2.5 | 60.00 | 20.41 | 17.67 |  |
|  | 100 |  | 3.5 | 3.33 | 4.17 | 0.33 |  |
|  | 100 |  | 4.5 | 0.33 | 33.33 | 0.33 |  |
|  | 100 |  | 5.5 | 0.00 | 0.00 | 0.00 |  |
|  | 100 |  | 6.5 | 32.67 | 31.96 | 31.00 |  |
|  | 100 |  | 7.5 | 12.00 | 66.67 | 12.00 |  |
| *Dipsacus strigosus* | 100 | 100 | 1.5 | 85.33 | 0.00 | 0.00 | long-term persistent |
|  | 100 |  | 2.5 | 82.00 | 0.79 | 0.67 |  |
|  | 100 |  | 3.5 | 78.33 | 1.39 | 1.00 |  |
|  | 100 |  | 4.5 | 84.33 | 0.00 | 0.00 |  |
|  | 100 |  | 5.5 | 88.67 | 0.75 | 0.67 |  |
|  | 100 |  | 6.5 | 54.67 | 1.23 | 1.00 |  |
|  | 100 |  | 7.5 | 89.67 | 14.80 | 13.00 |  |

**Supplementary Table S2.** Continued

| **species** | **no. buried seeds per sample bag** | **pre-burial viability (%)** | **years of burial** | **mean seed viability (% of buried seeds)** | **mean seed germinability (% of viable seeds)** | **mean seed germination (% of buried seeds)** | **seed bank persistence** |
| --- | --- | --- | --- | --- | --- | --- | --- |
| *Duchesnea indica* | 100 | 93 | 1.5 | 70.00 | 78.49 | 52.67 | long-term persistent |
|  | 100 |  | 2.5 | 49.67 | 98.78 | 48.67 |  |
|  | 100 |  | 3.5 | 17.33 | 56.62 | 11.67 |  |
|  | 100 |  | 4.5 | 29.00 | 39.78 | 15.00 |  |
|  | 100 |  | 5.5 | 20.33 | 38.89 | 16.00 |  |
|  | 100 |  | 6.5 | 11.00 | 3.70 | 0.33 |  |
|  | 100 |  | 7.5 | 34.33 | 96.75 | 33.67 |  |
| *Dysphania pumilio* | 100 | 85 | 1.5 | 41.67 | 100.00 | 41.67 | long-term persistent |
|  | 100 |  | 2.5 | 31.67 | 100.00 | 31.67 |  |
|  | 100 |  | 3.5 | 15.67 | 93.33 | 14.33 |  |
|  | 100 |  | 4.5 | 8.67 | 100.00 | 8.67 |  |
|  | 100 |  | 5.5 | 11.00 | 80.00 | 8.67 |  |
|  | 100 |  | 6.5 | 12.00 | 81.79 | 10.33 |  |
|  | 100 |  | 7.5 | 11.00 | 66.67 | 11.00 |  |
| *Echinocystis lobata* | 20 | 100 | 1.5 | 13.33 | 0.00 | 0.00 | long-term persistent |
|  | 20 |  | 2.5 | 15.00 | 0.00 | 0.00 |  |
|  | 20 |  | 3.5 | 31.67 | 0.00 | 0.00 |  |
|  | 20 |  | 4.5 | 28.33 | 5.56 | 1.67 |  |
|  | 20 |  | 5.5 | 15.00 | 0.00 | 0.00 |  |
|  | 20 |  | 6.5 | 10.00 | 16.67 | 1.67 |  |
|  | 20 |  | 7.5 | 8.33 | 11.11 | 1.67 |  |
| *Echinops sphaerocephalus* | 100 | 82 | 1.5 | 39.67 | 19.67 | 9.00 | long-term persistent |
|  | 100 |  | 2.5 | 38.00 | 11.42 | 3.67 |  |
|  | 100 |  | 3.5 | 37.33 | 71.77 | 23.67 |  |
|  | 100 |  | 4.5 | 12.67 | 87.50 | 10.67 |  |
|  | 100 |  | 5.5 | 5.33 | 64.44 | 5.00 |  |
|  | 100 |  | 6.5 | 5.00 | 63.89 | 4.67 |  |
|  | 100 |  | 7.5 | 12.00 | 66.67 | 12.00 |  |
| *Galega officinalis* | 100 | 100 | 1.5 | 87.00 | 32.15 | 26.67 | long-term persistent |
|  | 100 |  | 2.5 | 51.67 | 30.08 | 15.00 |  |
|  | 100 |  | 3.5 | 40.00 | 86.05 | 35.67 |  |
|  | 100 |  | 4.5 | 45.67 | 62.06 | 26.33 |  |
|  | 100 |  | 5.5 | 21.67 | 30.39 | 5.67 |  |
|  | 100 |  | 6.5 | 41.33 | 89.97 | 39.67 |  |
|  | 100 |  | 7.5 | 33.67 | 88.83 | 29.33 |  |
| *Galinsoga parviflora* | 100 | 100 | 1.5 | 62.00 | 100.00 | 62.00 | long-term persistent |
|  | 100 |  | 2.5 | 0.67 | 33.33 | 0.67 |  |
|  | 100 |  | 3.5 | 18.67 | 100.00 | 18.67 |  |
|  | 100 |  | 4.5 | 4.33 | 33.33 | 4.33 |  |
|  | 100 |  | 5.5 | 0.67 | 66.67 | 0.67 |  |
|  | 100 |  | 6.5 | 18.33 | 94.44 | 17.67 |  |
|  | 100 |  | 7.5 | 9.33 | 100.00 | 9.33 |  |
| *Heracleum mantegazzianum* | 100 | 82 | 1.5 | 1.00 | 33.33 | 0.67 | short-term persistent |
|  | 100 |  | 2.5 | 1.00 | 50.00 | 0.67 |  |
|  | 100 |  | 3.5 | 0.00 | 0.00 | 0.00 |  |
|  | 100 |  | 4.5 | 0.00 | 0.00 | 0.00 |  |
|  | 100 |  | 5.5 | 0.00 | 0.00 | 0.00 |  |
|  | 100 |  | 6.5 | 0.00 | 0.00 | 0.00 |  |
|  | 100 |  | 7.5 | 0.00 | 0.00 | 0.00 |  |
| *Hordeum jubatum* | 100 | 96 | 1.5 | 32.33 | 100.00 | 32.33 | short-term persistent |
|  | 100 |  | 2.5 | 1.00 | 33.33 | 1.00 |  |
|  | 100 |  | 3.5 | 0.00 | 0.00 | 0.00 |  |
|  | 100 |  | 4.5 | 0.00 | 0.00 | 0.00 |  |
|  | 100 |  | 5.5 | 0.00 | 0.00 | 0.00 |  |
|  | 100 |  | 6.5 | 0.00 | 0.00 | 0.00 |  |
|  | 100 |  | 7.5 | 0.00 | 0.00 | 0.00 |  |
| *Iva xanthiifolia* | 40 | 89 | 1.5 | 45.83 | 90.00 | 40.83 | long-term persistent |
|  | 40 |  | 2.5 | 30.83 | 66.67 | 25.83 |  |
|  | 40 |  | 3.5 | 21.67 | 72.22 | 17.50 |  |
|  | 40 |  | 4.5 | 20.00 | 100.00 | 20.00 |  |
|  | 40 |  | 5.5 | 12.50 | 75.56 | 9.17 |  |
|  | 40 |  | 6.5 | 7.50 | 100.00 | 7.50 |  |
|  | 40 |  | 7.5 | 21.67 | 100.00 | 21.67 |  |
| *Lepidium densiflorum* | 100 | 99 | 1.5 | 73.00 | 8.28 | 6.00 | long-term persistent |
|  | 100 |  | 2.5 | 52.00 | 5.69 | 3.00 |  |
|  | 100 |  | 3.5 | 46.33 | 1.54 | 1.00 |  |
|  | 100 |  | 4.5 | 34.33 | 79.27 | 27.00 |  |
|  | 100 |  | 5.5 | 16.67 | 3.40 | 1.67 |  |
|  | 100 |  | 6.5 | 12.33 | 55.67 | 3.67 |  |
|  | 100 |  | 7.5 | 38.33 | 54.49 | 18.67 |  |

**Supplementary Table S2.** Continued

| **species** | **no. buried seeds per sample bag** | **pre-burial viability (%)** | **years of burial** | **mean seed viability (% of buried seeds)** | **mean seed germinability (% of viable seeds)** | **mean seed germination (% of buried seeds)** | **seed bank persistence** |
| --- | --- | --- | --- | --- | --- | --- | --- |
| *Lupinus polyphyllus* | 100 | 93 | 1.5 | 29.67 | 3.41 | 1.00 | long-term persistent |
|  | 100 |  | 2.5 | 19.67 | 2.80 | 0.67 |  |
|  | 100 |  | 3.5 | 12.33 | 30.53 | 2.33 |  |
|  | 100 |  | 4.5 | 10.33 | 4.44 | 0.67 |  |
|  | 100 |  | 5.5 | 2.33 | 5.56 | 0.33 |  |
|  | 100 |  | 6.5 | 3.33 | 0.00 | 0.00 |  |
|  | 100 |  | 7.5 | 2.33 | 0.00 | 0.00 |  |
| *Matricaria discoidea* | 100 | 83 | 1.5 | 46.67 | 97.28 | 45.00 | long-term persistent |
|  | 100 |  | 2.5 | 20.00 | 94.44 | 18.67 |  |
|  | 100 |  | 3.5 | 17.33 | 98.25 | 17.00 |  |
|  | 100 |  | 4.5 | 20.00 | 100.00 | 20.00 |  |
|  | 100 |  | 5.5 | 2.00 | 66.67 | 2.00 |  |
|  | 100 |  | 6.5 | 10.00 | 100.00 | 10.00 |  |
|  | 100 |  | 7.5 | 5.67 | 33.33 | 5.67 |  |
| *Medicago sativa* | 100 | 88 | 1.5 | 29.67 | 24.39 | 7.33 | short-term persistent |
|  | 100 |  | 2.5 | 10.00 | 17.94 | 1.67 |  |
|  | 100 |  | 3.5 | 0.00 | 0.00 | 0.00 |  |
|  | 100 |  | 4.5 | 0.00 | 0.00 | 0.00 |  |
|  | 100 |  | 5.5 | 0.00 | 0.00 | 0.00 |  |
|  | 100 |  | 6.5 | 0.00 | 0.00 | 0.00 |  |
|  | 100 |  | 7.5 | 0.00 | 0.00 | 0.00 |  |
| *Myrrhis odorata* | 100 | 88 | 1.5 | 0.00 | 0.00 | 0.00 | transient |
|  | 100 |  | 2.5 | 0.00 | 0.00 | 0.00 |  |
|  | 100 |  | 3.5 | 0.00 | 0.00 | 0.00 |  |
|  | 100 |  | 4.5 | 0.00 | 0.00 | 0.00 |  |
|  | 100 |  | 5.5 | 0.00 | 0.00 | 0.00 |  |
|  | 100 |  | 6.5 | 0.00 | 0.00 | 0.00 |  |
|  | 100 |  | 7.5 | 0.00 | 0.00 | 0.00 |  |
| *Oenothera biennis* | 100 | 100 | 1.5 | 94.67 | 100.00 | 94.67 | long-term persistent |
|  | 100 |  | 2.5 | 74.33 | 100.00 | 74.33 |  |
|  | 100 |  | 3.5 | 65.33 | 100.00 | 65.33 |  |
|  | 100 |  | 4.5 | 35.00 | 100.00 | 35.00 |  |
|  | 100 |  | 5.5 | 65.00 | 99.60 | 64.67 |  |
|  | 100 |  | 6.5 | 45.33 | 100.00 | 45.33 |  |
|  | 100 |  | 7.5 | 54.67 | 100.00 | 54.67 |  |
| *Oenothera glazioviana* | 100 | 50 | 1.5 | 40.67 | 99.29 | 40.33 | long-term persistent |
|  | 100 |  | 2.5 | 42.00 | 100.00 | 42.00 |  |
|  | 100 |  | 3.5 | 27.67 | 98.99 | 27.33 |  |
|  | 100 |  | 4.5 | 31.33 | 100.00 | 31.33 |  |
|  | 100 |  | 5.5 | 31.00 | 98.29 | 30.33 |  |
|  | 100 |  | 6.5 | 23.67 | 100.00 | 23.67 |  |
|  | 100 |  | 7.5 | 12.33 | 100.00 | 12.33 |  |
| *Oxalis stricta* | 100 | 61 | 1.5 | 49.67 | 97.87 | 48.67 | long-term persistent |
|  | 100 |  | 2.5 | 41.33 | 99.32 | 41.00 |  |
|  | 100 |  | 3.5 | 27.33 | 87.16 | 23.67 |  |
|  | 100 |  | 4.5 | 35.00 | 100.00 | 35.00 |  |
|  | 100 |  | 5.5 | 29.33 | 82.86 | 24.67 |  |
|  | 100 |  | 6.5 | 36.33 | 91.46 | 33.33 |  |
|  | 100 |  | 7.5 | 46.00 | 97.16 | 44.67 |  |
| *Oxybaphus nyctagineus* | 100 | 95 | 1.5 | 74.67 | 100.00 | 74.67 | long-term persistent |
|  | 100 |  | 2.5 | 72.67 | 99.40 | 72.33 |  |
|  | 100 |  | 3.5 | 53.67 | 100.00 | 53.67 |  |
|  | 100 |  | 4.5 | 18.00 | 100.00 | 18.00 |  |
|  | 100 |  | 5.5 | 21.33 | 100.00 | 21.33 |  |
|  | 100 |  | 6.5 | 19.67 | 100.00 | 19.67 |  |
|  | 100 |  | 7.5 | 13.33 | 66.67 | 13.33 |  |
| *Panicum capillare* | 100 | 78 | 1.5 | 50.33 | 93.33 | 49.00 | long-term persistent |
|  | 100 |  | 2.5 | 73.67 | 100.00 | 73.67 |  |
|  | 100 |  | 3.5 | 45.33 | 88.89 | 37.00 |  |
|  | 100 |  | 4.5 | 54.67 | 66.67 | 54.67 |  |
|  | 100 |  | 5.5 | 5.67 | 100.00 | 5.67 |  |
|  | 100 |  | 6.5 | 20.00 | 100.00 | 20.00 |  |
|  | 100 |  | 7.5 | 19.67 | 65.43 | 19.00 |  |
| *Panicum miliaceum* | 100 | 99 | 1.5 | 85.33 | 29.09 | 25.33 | long-term persistent |
|  | 100 |  | 2.5 | 59.00 | 50.40 | 32.67 |  |
|  | 100 |  | 3.5 | 38.33 | 11.24 | 9.33 |  |
|  | 100 |  | 4.5 | 29.00 | 30.65 | 26.67 |  |
|  | 100 |  | 5.5 | 8.33 | 6.06 | 0.67 |  |
|  | 100 |  | 6.5 | 21.67 | 55.17 | 8.00 |  |
|  | 100 |  | 7.5 | 8.67 | 21.79 | 5.67 |  |

**Supplementary Table S2.** Continued

| **species** | **no. buried seeds per sample bag** | **pre-burial viability (%)** | **years of burial** | **mean seed viability (% of buried seeds)** | **mean seed germinability (% of viable seeds)** | **mean seed germination (% of buried seeds)** | **seed bank persistence** |
| --- | --- | --- | --- | --- | --- | --- | --- |
| *Phytolacca esculenta* | 100 | 98 | 1.5 | 74.00 | 73.74 | 54.00 | long-term persistent |
|  | 100 |  | 2.5 | 73.67 | 99.56 | 73.33 |  |
|  | 100 |  | 3.5 | 63.00 | 97.09 | 61.33 |  |
|  | 100 |  | 4.5 | 63.67 | 66.51 | 40.00 |  |
|  | 100 |  | 5.5 | 68.00 | 83.00 | 58.00 |  |
|  | 100 |  | 6.5 | 29.00 | 91.53 | 28.00 |  |
|  | 100 |  | 7.5 | 53.33 | 40.29 | 17.67 |  |
| *Potentilla intermedia* | 100 | 63 | 1.5 | 43.00 | 69.25 | 29.67 | long-term persistent |
|  | 100 |  | 2.5 | 46.33 | 99.29 | 46.00 |  |
|  | 100 |  | 3.5 | 35.33 | 94.98 | 33.67 |  |
|  | 100 |  | 4.5 | 14.67 | 100.00 | 14.67 |  |
|  | 100 |  | 5.5 | 12.33 | 100.00 | 12.33 |  |
|  | 100 |  | 6.5 | 16.00 | 97.98 | 15.33 |  |
|  | 100 |  | 7.5 | 18.00 | 100.00 | 18.00 |  |
| *Pseudofumaria lutea* | 100 | 79 | 1.5 | 8.67 | 33.33 | 4.67 | short-term persistent |
|  | 100 |  | 2.5 | 0.00 | 0.00 | 0.00 |  |
|  | 100 |  | 3.5 | 0.00 | 0.00 | 0.00 |  |
|  | 100 |  | 4.5 | 0.00 | 0.00 | 0.00 |  |
|  | 100 |  | 5.5 | 0.00 | 0.00 | 0.00 |  |
|  | 100 |  | 6.5 | 0.00 | 0.00 | 0.00 |  |
|  | 100 |  | 7.5 | 0.00 | 0.00 | 0.00 |  |
| *Rudbeckia hirta* | 50 | 59 | 1.5 | 61.33 | 85.56 | 55.33 | long-term persistent |
|  | 50 |  | 2.5 | 24.67 | 100.00 | 24.67 |  |
|  | 50 |  | 3.5 | 20.67 | 83.33 | 20.00 |  |
|  | 50 |  | 4.5 | 5.33 | 33.33 | 5.33 |  |
|  | 50 |  | 5.5 | 1.33 | 66.67 | 1.33 |  |
|  | 50 |  | 6.5 | 6.67 | 66.67 | 6.67 |  |
|  | 50 |  | 7.5 | 10.67 | 66.67 | 10.67 |  |
| *Rudbeckia laciniata* | 100 | 85 | 1.5 | 76.67 | 100.00 | 76.67 | short-term persistent |
|  | 100 |  | 2.5 | 51.00 | 66.67 | 51.00 |  |
|  | 100 |  | 3.5 | 27.67 | 66.67 | 27.67 |  |
|  | 100 |  | 4.5 | 1.00 | 66.67 | 1.00 |  |
|  | 100 |  | 5.5 | 3.67 | 33.33 | 3.67 |  |
|  | 100 |  | 6.5 | 0.33 | 33.33 | 0.33 |  |
|  | 100 |  | 7.5 | 0.00 | 0.00 | 0.00 |  |
| *Rumex alpinus* | 100 | 85 | 1.5 | 73.33 | 100.00 | 73.33 | long-term persistent |
|  | 100 |  | 2.5 | 45.00 | 100.00 | 45.00 |  |
|  | 100 |  | 3.5 | 51.00 | 65.77 | 50.33 |  |
|  | 100 |  | 4.5 | 54.67 | 100.00 | 54.67 |  |
|  | 100 |  | 5.5 | 49.00 | 100.00 | 49.00 |  |
|  | 100 |  | 6.5 | 57.33 | 100.00 | 57.33 |  |
|  | 100 |  | 7.5 | 14.00 | 100.00 | 14.00 |  |
| *Rumex longifolius* | 100 | 100 | 1.5 | 92.00 | 100.00 | 92.00 | long-term persistent |
|  | 100 |  | 2.5 | 91.33 | 100.00 | 91.33 |  |
|  | 100 |  | 3.5 | 67.67 | 100.00 | 67.67 |  |
|  | 100 |  | 4.5 | 50.67 | 66.67 | 50.67 |  |
|  | 100 |  | 5.5 | 31.00 | 99.27 | 30.33 |  |
|  | 100 |  | 6.5 | 19.33 | 100.00 | 19.33 |  |
|  | 100 |  | 7.5 | 0.00 | 0.00 | 0.00 |  |
| *Rumex patientia* | 100 | 93 | 1.5 | 41.00 | 100.00 | 41.00 | long-term persistent |
|  | 100 |  | 2.5 | 13.00 | 100.00 | 13.00 |  |
|  | 100 |  | 3.5 | 16.67 | 65.08 | 16.33 |  |
|  | 100 |  | 4.5 | 19.33 | 33.33 | 19.33 |  |
|  | 100 |  | 5.5 | 2.00 | 33.33 | 2.00 |  |
|  | 100 |  | 6.5 | 28.67 | 100.00 | 28.67 |  |
|  | 100 |  | 7.5 | 3.67 | 66.67 | 3.67 |  |
| *Rumex thyrsiflorus* | 100 | 61 | 1.5 | 1.00 | 100.00 | 1.00 | short-term persistent |
|  | 100 |  | 2.5 | 1.33 | 33.33 | 1.33 |  |
|  | 100 |  | 3.5 | 0.00 | 0.00 | 0.00 |  |
|  | 100 |  | 4.5 | 0.67 | 66.67 | 0.67 |  |
|  | 100 |  | 5.5 | 0.33 | 0.00 | 0.00 |  |
|  | 100 |  | 6.5 | 0.33 | 33.33 | 0.33 |  |
|  | 100 |  | 7.5 | 0.00 | 0.00 | 0.00 |  |
| *Scutellaria altissima* | 100 | 81 | 1.5 | 18.00 | 4.94 | 2.67 | short-term persistent |
|  | 100 |  | 2.5 | 1.00 | 11.11 | 0.33 |  |
|  | 100 |  | 3.5 | 0.00 | 0.00 | 0.00 |  |
|  | 100 |  | 4.5 | 0.33 | 33.33 | 0.33 |  |
|  | 100 |  | 5.5 | 0.00 | 0.00 | 0.00 |  |
|  | 100 |  | 6.5 | 0.00 | 0.00 | 0.00 |  |
|  | 100 |  | 7.5 | 0.00 | 0.00 | 0.00 |  |

**Supplementary Table S2.** Continued

| **species** | **no. buried seeds per sample bag** | **pre-burial viability (%)** | **years of burial** | **mean seed viability (% of buried seeds)** | **mean seed germinability (% of viable seeds)** | **mean seed germination (% of buried seeds)** | **seed bank persistence** |
| --- | --- | --- | --- | --- | --- | --- | --- |
| *Senecio leucanthemifolius* | 100 | 92 | 1.5 | 0.33 | 0.00 | 0.00 | short-term persistent |
| subsp. *vernalis* | 100 |  | 2.5 | 0.00 | 0.00 | 0.00 |  |
|  | 100 |  | 3.5 | 0.00 | 0.00 | 0.00 |  |
|  | 100 |  | 4.5 | 0.67 | 66.67 | 0.67 |  |
|  | 100 |  | 5.5 | 0.00 | 0.00 | 0.00 |  |
|  | 100 |  | 6.5 | 0.33 | 0.00 | 0.00 |  |
|  | 100 |  | 7.5 | 0.00 | 0.00 | 0.00 |  |
| *Setaria faberi* | 100 | 94 | 1.5 | 61.67 | 91.20 | 56.33 | long-term persistent |
|  | 100 |  | 2.5 | 17.00 | 95.45 | 16.00 |  |
|  | 100 |  | 3.5 | 21.33 | 66.67 | 21.33 |  |
|  | 100 |  | 4.5 | 8.00 | 33.33 | 8.00 |  |
|  | 100 |  | 5.5 | 0.00 | 0.00 | 0.00 |  |
|  | 100 |  | 6.5 | 9.00 | 66.67 | 9.00 |  |
|  | 100 |  | 7.5 | 11.00 | 100.00 | 11.00 |  |
| *Sisymbrium loeselii* | 100 | 81 | 1.5 | 51.00 | 50.97 | 28.33 | long-term persistent |
|  | 100 |  | 2.5 | 36.33 | 81.37 | 31.00 |  |
|  | 100 |  | 3.5 | 27.67 | 72.52 | 20.33 |  |
|  | 100 |  | 4.5 | 16.67 | 100.00 | 16.67 |  |
|  | 100 |  | 5.5 | 14.33 | 55.83 | 10.00 |  |
|  | 100 |  | 6.5 | 11.67 | 76.59 | 8.67 |  |
|  | 100 |  | 7.5 | 20.00 | 88.77 | 16.67 |  |
| *Sisymbrium strictissimum* | 100 | 99 | 1.5 | 54.67 | 100.00 | 54.67 | long-term persistent |
|  | 100 |  | 2.5 | 28.67 | 76.19 | 27.00 |  |
|  | 100 |  | 3.5 | 25.67 | 95.24 | 25.33 |  |
|  | 100 |  | 4.5 | 24.67 | 100.00 | 24.67 |  |
|  | 100 |  | 5.5 | 4.33 | 61.90 | 2.33 |  |
|  | 100 |  | 6.5 | 2.67 | 100.00 | 2.67 |  |
|  | 100 |  | 7.5 | 8.67 | 66.67 | 8.67 |  |
| *Smyrnium perfoliatum* | 100 | 89 | 1.5 | 0.00 | 0.00 | 0.00 | transient |
|  | 100 |  | 2.5 | 0.00 | 0.00 | 0.00 |  |
|  | 100 |  | 3.5 | 0.00 | 0.00 | 0.00 |  |
|  | 100 |  | 4.5 | 0.00 | 0.00 | 0.00 |  |
|  | 100 |  | 5.5 | 0.00 | 0.00 | 0.00 |  |
|  | 100 |  | 6.5 | 0.00 | 0.00 | 0.00 |  |
|  | 100 |  | 7.5 | 0.00 | 0.00 | 0.00 |  |
| *Solidago canadensis* | 100 | 63 | 1.5 | 26.33 | 82.68 | 25.67 | long-term persistent |
|  | 100 |  | 2.5 | 10.67 | 100.00 | 10.67 |  |
|  | 100 |  | 3.5 | 3.67 | 83.33 | 3.00 |  |
|  | 100 |  | 4.5 | 2.33 | 66.67 | 2.33 |  |
|  | 100 |  | 5.5 | 2.67 | 100.00 | 2.67 |  |
|  | 100 |  | 6.5 | 2.33 | 100.00 | 2.33 |  |
|  | 100 |  | 7.5 | 1.33 | 66.67 | 1.33 |  |
| *Solidago gigantea* | 100 | 95 | 1.5 | 26.00 | 100.00 | 26.00 | long-term persistent |
|  | 100 |  | 2.5 | 2.00 | 100.00 | 2.00 |  |
|  | 100 |  | 3.5 | 6.33 | 66.67 | 6.33 |  |
|  | 100 |  | 4.5 | 0.67 | 66.67 | 0.67 |  |
|  | 100 |  | 5.5 | 0.00 | 0.00 | 0.00 |  |
|  | 100 |  | 6.5 | 1.33 | 66.67 | 1.33 |  |
|  | 100 |  | 7.5 | 1.67 | 100.00 | 1.67 |  |
| *Telekia speciosa* | 100 | 48 | 1.5 | 33.67 | 99.52 | 33.33 | long-term persistent |
|  | 100 |  | 2.5 | 0.33 | 33.33 | 0.33 |  |
|  | 100 |  | 3.5 | 0.67 | 16.67 | 0.33 |  |
|  | 100 |  | 4.5 | 0.00 | 0.00 | 0.00 |  |
|  | 100 |  | 5.5 | 0.00 | 0.00 | 0.00 |  |
|  | 100 |  | 6.5 | 0.00 | 0.00 | 0.00 |  |
|  | 100 |  | 7.5 | 1.00 | 66.67 | 1.00 |  |
| *Xanthium albinum* | 100 | 72 | 1.5 | 41.00 | 68.67 | 29.00 | long-term persistent |
|  | 100 |  | 2.5 | 29.67 | 85.79 | 26.00 |  |
|  | 100 |  | 3.5 | 31.67 | 95.13 | 30.00 |  |
|  | 100 |  | 4.5 | 13.00 | 66.67 | 13.00 |  |
|  | 100 |  | 5.5 | 0.00 | 0.00 | 0.00 |  |
|  | 100 |  | 6.5 | 2.00 | 66.67 | 2.00 |  |
|  | 100 |  | 7.5 | 10.67 | 66.67 | 10.67 |  |

References

Thompson, K., Bakker, J. P., & Bekker, R. M. *Soil Seed Banks of NW Europe: Methodology, Density and Longevity*. (Cambridge University Press, Cambridge, 1997).

**Supplementary Table S3.** Results of logistic phylogenetic models, modelling (a) seed viability percentage and (b) seed germinability percentage as functions of time (number of years since burial) and its interaction with invasiveness (invasive species (21 species) versus naturalized but non-invasive in the Czech Republic (38 species), seed mass [log(x)] (g), life form (annual versus perennial herbs), seed bank type (transient versus persistent, from GloSSBank; Gioria et al. 2020).

| **model** | ***R*^2^** | **posterior mean** | **lower 95% C.I.** | **upper 95% C.I.** | ***p*MCMC** | **phylogeny** | **species** | **triplets** | **row sequence** | **row number** | **units** |
| --- | --- | --- | --- | --- | --- | --- | --- | --- | --- | --- | --- |
| **a) seed viability percentage** | |  |  |  |  |  |  |  |  |  |  |
| time:invasiveness | 0.974 | -0.23 | -0.353 | -0.109 | 0 | 18.870 (6.415 - 31.990) | 1.223 (0.000 - 4.129) | 95.884 (0.860 - 265.002) | 0.113 (0.000 - 0.276) | 0.210 (0.015 - 0.519) | 3.180 (2.840 - 3.547) |
| time:log[seed mass] | 0.973 | -0.131 | -0.252 | -0.006 | 0.038 | 19.428 (6.769 - 33.265) | 1.193 (0.000 - 4.180) | 93.501 (0.966 - 249.938) | 0.113 (0.000 - 0.277) | 0.210 (0.015 - 0.534) | 3.216 (2.872 - 3.584) |
| time:life form | 0.973 | 0.054 | -0.07 | 0.18 | 0.398 | 18.807 (6.033 - 32.491) | 1.304 (0.000 - 4.377) | 94.086 (0.898 - 250.238) | 0.116 (0.000 - 0.289) | 0.213 (0.010 - 0.520) | 3.223 (2.883 - 3.601) |
| time:seed bank type | 0.972 | -0.021 | -0.146 | 0.103 | 0.747 | 19.120 (6.532 - 33.354) | 1.248 (0.000 - 4.307) | 90.857 (0.658 - 242.567) | 0.115 (0.000 - 0.289) | 0.208 (0.016 - 0.521) | 3.229 (2.877 - 3.586) |
| time | 0.973 | -4.871 | -11.606 | 0.351 | 0.049 | 19.295 (5.597 - 32.581) | 1.235 (0.000 - 4.227) | 69.825 (0.001 - 214.725) | 0.117 (0.000 - 0.289) | 0.207 (0.009 - 0.516) | 3.226 (2.873 - 3.585) |
| **b) seed germinability percentage** | |  |  |  |  |  |  |  |  |  |  |
| time:invasiveness | 0.868 | 0.005 | -0.203 | 0.206 | 0.952 | 18.896 (6.502 - 33.913) | 3.882 (0.429 - 8.447) | 2.094 (0.194 - 5.807) | 0.040 (0.000 - 0.156) | 0.068 (0.000 - 0.263) | 3.994 (3.270 - 4.767) |
| time:log[seed mass] | 0.867 | 0.152 | -0.036 | 0.334 | 0.109 | 18.789 (6.754 - 34.479) | 3.773 (0.380 - 8.224) | 2.012 (0.190 - 5.543) | 0.039 (0.000 - 0.155) | 0.063 (0.000 - 0.247) | 3.964 (3.246 - 4.745) |
| time:life form | 0.867 | -0.002 | -0.206 | 0.192 | 0.986 | 18.992 (6.064 - 34.040) | 3.769 (0.385 - 8.190) | 2.046 (0.201 - 5.613) | 0.039 (0.000 - 0.154) | 0.066 (0.000 - 0.257) | 3.994 (3.295 - 4.796) |
| time:seed bank type | 0.867 | -0.042 | -0.215 | 0.128 | 0.639 | 18.931 (5.791 - 33.080) | 3.790 (0.458 - 8.267) | 2.012 (0.192 - 5.383) | 0.039 (0.000 - 0.154) | 0.067 (0.000 - 0.252) | 3.998 (3.269 - 4.767) |
| time | 0.871 | 0.374 | -0.848 | 1.597 | 0.438 | 18.988 (6.645 - 33.691) | 3.748 (0.244 - 7.956) | 2.661 (0.163 - 7.561) | 0.039 (0.000 - 0.153) | 0.068 (0.000 - 0.265) | 3.983 (3.236 - 4.728) |

References

Gioria, M., Pyšek, P., Baskin, C., & Carta, A. Phylogenetic relatedness mediates persistence and density of soil seed banks. *J Ecol.* **108**, 2121–2131 (2020).

**Supplementary Table S4.** Phylogenetic signal (λ) in (a) seed viability percentage and (b) seed germinability percentage, based on data collected for 59 alien herbaceous species in the Czech Republic.

| **Models** | **lambda** | **lower values** | **upper values** |
| --- | --- | --- | --- |
| **a. seed viability percentage** |  |  |  |
| time:invasiveness | 0.84 | 0.727 | 0.926 |
| time:log[seed mass] | 0.842 | 0.733 | 0.927 |
| time:life form | 0.836 | 0.715 | 0.927 |
| time:seed bank type | 0.839 | 0.72 | 0.925 |
| time | 0.84 | 0.721 | 0.928 |
| **b. seed germinability percentage** |  |  |  |
| time:invasiveness | 0.807 | 0.671 | 0.91 |
| time:log[seed mass] | 0.808 | 0.681 | 0.915 |
| time:life form | 0.808 | 0.682 | 0.916 |
| time:seed bank type | 0.808 | 0.679 | 0.913 |
| time | 0.809 | 0.688 | 0.913 |

**Supplementary Table S5.** List of localities where seeds of 59 species used in the burial experiment had been collected from, together with a description of the habitats characterizing the sampled populations and geographic coordinates, and date of seed collection.

| **species ID** | **Species** | **locality** | **district** | **locality description** | **latitude** | **longitude** | **date of collection** |
| --- | --- | --- | --- | --- | --- | --- | --- |
| 1 | *Abutilon theophrasti* | Mutěnice | Hodonín | dump on the east edge of Srálkovský pond | 48°54'26.3'' | 17°03'13.9'' | 01/10/2012 |
| 2 | *Amaranthus albus* | Břeclav | Břeclav | railway bank at the railway station Břeclav-zastávka | 48°45'47.4'' | 16°54'10.4'' | 30/09/2012 |
| 2 | *Amaranthus albus* | Beroun | Beroun | railway station Beroun | 49°57'23.4'' | 14°04'22.3'' | 17/10/2012 |
| 3 | *Amaranthus powellii* | Dobřichovice | Praha-západ | ruderal site near the Berounka river in the village | 49°55'46.3'' | 14°17'15.1'' | 17/10/2012 |
| 3 | *Amaranthus powellii* | Břeclav | Břeclav | field edge close to the parking place, 1.2 km SSE of the railway station Břeclav | 48°44'35.8'' | 16°53'54.4'' | 30/09/2012 |
| 4 | *Amaranthus retroflexus* | Lanžhot | Břeclav | ruderal site close to soil waste site, 1.4 km SE of the village | 48°43'39,5'' | 16°58'37.0'' | 01/10/2012 |
| 4 | *Amaranthus retroflexus* | Sedlec u Mikulova | Břeclav | field edge 1,4km NE of theSedlec church | 48°47'26,2'' | 16°42'20.5'' | 28/09/2012 |
| 4 | *Amaranthus retroflexus* | Černošice | Praha-západ | field edge 1,7km NE of the Černošice railway station | 49°57'53,8'' | 14°20'24.6'' | 17/10/2012 |
| 5 | *Ambrosia artemisiifolia* | Břeclav | Břeclav | field edge close to the parking place Pohansko, 1.2 km SSE of the railway station Břeclav | 48°44'32.8'' | 16°53'49.8'' | 30/09/2012 |
| 5 | *Ambrosia artemisiifolia* | Lanžhot | Břeclav | ruderal site close to soil waste site, 1.4 km SE of the village | 48°43'35.0'' | 16°58'42.7'' | 30/09/2012 |
| 5 | *Ambrosia artemisiifolia* | Pečky | Kolín | railway station Pečky | 50°05'29.0'' | 15°01'14.1'' | 23/10/2012 |
| 6 | *Ambrosia trifida* | Veltruby | Kolín | edge of the field, 0.9 km SSW of the railway station Veltruby | 50°04'12.8'' | 15°11'25.9'' | 20/09/2012 |
| 7 | *Antirrhinum majus* | Mikulov | Břeclav | walls of the Mikulov castle | 48°48'25.8'' | 16°38'13.2'' | 15/07/2012 |
| 7 | *Antirrhinum majus* | Štramberk | Nový Jičín | walls of the Štramberk houses in the village | 49°35'15.6'' | 18°07'22.4'' | 12/07/2012 |
| 8 | *Arabis alpina* | Mikulov | Břeclav | walls of the Mikulov castle | 48°48'22.6'' | 16°38'09.2'' | 15/07/2012 |
| 8 | *Arabis alpina* | Velhartice | Klatovy | walls of the Velhartice castle | 49°15'46.9'' | 13°23'59.0'' | 05/08/2012 |
| 9 | *Asclepias syriaca* | Břeclav | Břeclav | meadow close to Pohansko castle | 48°43'50.6'' | 16°53'45.6' | 30/09/2012 |
| 9 | *Asclepias syriaca* | Břeclav-Poštorná | Břeclav | road bank, 0.5 km WNW of the railway station Boří les | 48°44'19.5'' | 16°51'44.6' | 01/10/2012 |
| 10 | *Bassia scoparia* | Praha-Radlice | Praha | road edge of the Strakonická street, 1.1 km SSE of the railway station Praha-Smíchov | 50°03'04.9'' | 14°24'37.7'' | 15/10/2012 |
| 10 | *Bassia scoparia* | Pečky | Kolín | railway station Pečky | 50°05'28.9'' | 15°01'15.5'' | 23/10/2012 |
| 11 | *Bidens connata* | Všestudy | Mělník | wetland near Všestudský potok brook, 0.5 km SSE of the Všestudy village municipality | 50°17'13.4'' | 14°20'33.2'' | 24/10/2012 |
| 12 | *Bidens frondosa* | Volary | Prachatice | brook side of Volarský potok 0,2 km fro the Volary church | 48°54'33.1'' | 13°53'36.5'' | 17/10/2012 |
| 12 | *Bidens frondosa* | Nová Ves I, Klavary | Kolín | Labe river bank, 1.9 km NE of the Nová Ves I church | 49°56'21.0'' | 14°19'27.4'' | 20/09/2012 |
| 12 | *Bidens frondosa* | Praha-Modřany | Praha | Vltava river bank, 0.3 km SSW of railway station of Praha-Modřany | 50°00'09.5'' | 14°24'10.1'' | 26/09/2012 |
| 13 | *Bunias orientalis* | Beroun-Jarov | Beroun | road bank, 1.7 km SSW of the railway station Beroun | 49°56'33.9'' | 14°04'03.2'' | 22/08/2012 |
| 13 | *Bunias orientalis* | Hostinné | Hostinné | road bank, 0.3 km W of railway station Prosečné | 50°32'54.4'' | 15°41'28.2'' | 16/08/2012 |
| 14 | *Cannabis sativa* | Čejkovice | Hodonín | field edge, 3.2 km ENE of the Čejkovice church | 48°54'27.7'' | 16°59'06.1'' | 01/10/2012 |
| 14 | *Cannabis sativa* | Sedlec | Břeclav | field edge, 1.8 km NNE of the railway station Sedlec | 48°47'22.7'' | 16°42'26.6'' | 29/09/2012 |
| 14 | *Cannabis sativa* | Drnholec | Břeclav | field edge, 1.8 km ESE of the Drnholec church | 48°51'14.0'' | 16°29'52.1'' | 29/09/2012 |
| 15 | *Claytonia sibirica* | Průhonice | Praha-západ | forest in the Park Průhonice | 49°59'51.9'' | 14°33'40.6'' | 26/07/2012 |
| 16 | *Consolida orientalis* | Trubín | Beroun | field edge, 1.1 km ESE of the Trubín village municipality | 49°56'28.3'' | 14°01'00.1'' | 22/07/2012 |
| 16 | *Consolida orientalis* | Lochkov | Praha | field edge, 0.5 km WNW of the Lochkov castle | 50°00'11.8'' | 14°21'40.0'' | 02/08/2012 |
| 16 | *Consolida orientalis* | Slivenec | Praha | field edge, 0.5 km NW of the Slivenec church | 50°01'12.7" | 14°20'51.8" | 02/08/2012 |

**Supplementary Table S5.** Continued

| **species ID** | **Species** | **locality** | **district** | **locality description** | **latitude** | **longitude** | **date of collection** |
| --- | --- | --- | --- | --- | --- | --- | --- |
| 17 | *Erigeron canadensis* | Pečky | Kolín | railway station Pečky | 50°05'28.9'' | 15°07'41.5'' | 10/09/2012 |
| 17 | *Erigeron canadensis* | Praha-Modřany | Praha | road edge, 0.5 km S of the railway station Praha-Modřany | 49°59'53.7" | 14°24'13.4" | 24/08/2012 |
| 17 | *Erigeron canadensis* | Týniště nad Orlicí | Rychnov nad Kněžnou | railway station Týniště nad Orlicí | 50°09'34.1" | 16°04'09.7" | 16/08/2012 |
| 18 | *Datura stramonium* | Sedlec | Břeclav | field edge, 0.8 km SE of the railway station Sedlec | 48°46'16.8'' | 16°42'33.7'' | 29/09/2012 |
| 18 | *Datura stramonium* | Hrušky | Břeclav | field edge, 1.8 km WSW of the railway station Hrušky | 48°46'36.2'' | 16°55'12.7'' | 30/09/2012 |
| 18 | *Datura stramonium* | Černošice | Praha-západ | field edge 2,2 km ENE of the railway station Černošice | 49°57'38.8'' | 14°20'40.1'' | 17/10/2012 |
| 19 | *Dipsacus strigosus* | Slivenec | Praha | road edge, | 50°01'12.7" | 14°20'51.8" | 22/08/2012 |
| 19 | *Dipsacus strigosus* | Praha-Cholupice | Praha | road edge at the NW edge of Cholupická bažantnice pheasantry natural monument | 49°59'05.8'' | 14°27'17.8'' | 25/08/2012 |
| 19 | *Dipsacus strigosus* | Srbsko | Beroun | road edge, 2 km ESE of the railway station Srbsko | 49°55'55.1'' | 14°09'20.9'' | 22/08/2012 |
| 20 | *Duchesnea indica* | Roztoky | Praha-západ | road bank, 0.3 km NW of the railway station Roztoky | 50°09'40.3'' | 14°23'42.4'' | 29/06/2012 |
| 20 | *Duchesnea indica* | Praha-Albertov | Praha | lawn along the road | 50°04'11.1'' | 14°25'36.7'' | 03/07/2012 |
| 20 | *Duchesnea indica* | Průhonice | Praha-západ | Park Průhonice | 50°00'03.2'' | 14°33'24.0'' | 23/08/2012 |
| 21 | *Dysphania pumilio* | Břeclav-Poštorná | Břeclav | pine forest, 1.3 km W of the railway station Břeclav-Poštorná | 48°45'06.6'' | 16°50'08.0'' | 28/09/2012 |
| 22 | *Echinocystis lobata* | Všenory | Praha-západ | Vltava river bank, 1.6 km ENE of the railway station Všenory | 49°56'18.7'' | 14°19'33.4'' | 02/10/2012 |
| 23 | *Echinops sphaerocephalus* | Velký Osek | Kolín | road edge, 1.3 km SW of the railway station Velký Osek | 50°05'38.0'' | 15°10'48.9'' | 10/09/2012 |
| 23 | *Echinops sphaerocephalus* | Hnanice | Znojmo | road edge, 0.7 km NEN of the Hnanice church | 48°48'11.2'' | 15°59'20.1'' | 29/09/2012 |
| 23 | *Echinops sphaerocephalus* | Praha-Modřany | Praha | slope of the railway station Praha Modřany | 50°00'12.8" | 14°24'10.5" | 09/09/2012 |
| 24 | *Galega officinalis* | Libice nad Cidlinou | Kolín | road edge, 3.5 km NW of the railway station Velký Osek | 50°07'03.4'' | 15°09'03.8'' | 10/09/2012 |
| 24 | *Galega officinalis* | Průhonice | Praha-západ | ruderal road edge in the village | 50°00'20.0'' | 14°33'56.0'' | 05/09/2012 |
| 25 | *Galinsoga parviflora* | Lanžhot | Břeclav | field edge, 1.2 km SW of the railway station Lanžhot | 48°43'33.8'' | 16°56'56.7'' | 30/09/2012 |
| 25 | *Galinsoga parviflora* | Všestudy | Mělník | edge of the road | 50°17'27.9'' | 14°20'16.2'' | 24/10/2012 |
| 25 | *Galinsoga parviflora* | Lysá nad Labem | Nymburk | field edge, 2.3 km NW of the railway station Lysá nad Labem | 50°12'56.9'' | 14°49'35.4'' | 10/09/2012 |
| 26 | *Heracleum mantegazzianum* | Lazy | Sokolov | meadow, 7.5 km N of the railway station Kynžvart | 50°03'45.2'' | 12°37'34.1'' | 03/09/2012 |
| 26 | *Heracleum mantegazzianum* | Mníšek pod Brdy | Praha-západ | ruderal site, 3.4 km NNE of the railway station Mníšek pod Brdy | 49°53'02.4'' | 14°16'47.7'' | 27/08/2012 |
| 27 | *Hordeum jubatum* | Zbraslav | Praha | ruderal site, 1,9 km from Zbraslavské náměstí square | 49°57'45.5'' | 14°22'47.6'' | 04/08/2012 |
| 27 | *Hordeum jubatum* | Razice- Radovesická výsypka | Teplice | ruderal site | 50°32'20.2'' | 13°48'55.4'' | 02/08/2012 |
| 27 | *Hordeum jubatum* | Praha-Kobylisy | Praha | road edge, 0.2 km NW of the Kobylisy-vozovna tram station | 50°07'44.8" | 14°31'21.3" | 28/09/2012 |
| 28 | *Iva xanthiifolia* | Beroun | Beroun | railway station Beroun | 49°57'23.4'' | 14°04'22.3'' | 10/10/2012 |
| 28 | *Iva xanthiifolia* | Liteň | Beroun | railway station Liteň | 49°54'11.3'' | 14°09'23.8'' | 11/10/2012 |
| 29 | *Lepidium densiflorum* | Strakonice | Strakonice | railway station Strakonice | 49°59'31.5'' | 14°24'06.6'' | 25/09/2012 |
| 29 | *Lepidium densiflorum* | Praha-Braník | Praha | railway station Praha-Braník | 50°01'44.5'' | 14°24'28.6'' | 18/07/2012 |
| 29 | *Lepidium densiflorum* | Pečky | Kolín | railway station Pečky | 50°05'28.4'' | 15°01'19.0'' | 06/09/2012 |
| 30 | *Lupinus polyphyllus* | Koněprusy | Beroun | limestone quarry Kobyla, 1.4 km SE of the Koněprusy church | 49°55'04.0" | 14°02'04.6" | 17/08/2012 |
| 30 | *Lupinus polyphyllus* | Pec pod sněžkou | Trutnov | meadow, 1.4 km NNW of the Pec pod Sněžkou church | 50°41'28.6'' | 15°42'54.1'' | 20/08/2012 |
| 30 | *Lupinus polyphyllus* | Borová Lada | Prachatice | road edge, 5.2 km SSW of the Borová Lada village | 48°57'05.2'' | 13°38'37.4'' | 25/10/2012 |
| 31 | *Matricaria discoidea* | Praha-Modřany | Praha | road edge, Vokrojova street in the town | 50°00'05.4'' | 14°25'42.5'' | 10/07/2012 |
| 31 | *Matricaria discoidea* | Kopřivnice | Nový Jičín | road edge, 0.2 km SSE of the railway station Kopřivnice | 49°35'36.0'' | 18°08'50.8'' | 12/07/2012 |

**Supplementary Table S5.** Continued

| **species ID** | **Species** | **locality** | **district** | **locality description** | **latitude** | **longitude** | **date of collection** |
| --- | --- | --- | --- | --- | --- | --- | --- |
| 32 | *Medicago sativa* | Praha-Modřany | Praha | road edge, Generála Šišky street, 1 km E of the railway station Praha-Modřany | 50°00'12.7'' | 14°24'59.2'' | 04/11/2012 |
| 32 | *Medicago sativa* | Praha-Barandov | Praha | road bank at K Barandovu street, SSW of the railway station Praha- Hlubočepy | 50°01'46.6'' | 14°22'48.5'' | 01/11/2012 |
| 33 | *Myrrhis odorata* | Velká Úpa | Trutnov | road bank, 0.5 km NE of the Velká Úpa church | 50°41'34.2'' | 15°46'46.0'' | 30/08/2012 |
| 34 | *Oenothera biennis* | Praha-Modřany | Praha | road edge, U kina street | 50°00'13.1'' | 14°24'13.3'' | 15/09/2012 |
| 34 | *Oenothera biennis* | Velký Osek | Kolín | road edge, 4 km NW of the railway station Velký Osek | 50°07'02.5'' | 15°08'27.8'' | 20/09/2012 |
| 35 | *Oenothera glazioviana* | Černolice | Praha-západ | road edge at the summerhouse area, 1.7 km SSE of the railway station Dobřichovice | 49°54'47.7'' | 14°17'58.7'' | 23/09/2012 |
| 35 | *Oenothera glazioviana* | Praha-Modřany | Praha | ruderal site, 0,6 kmNE from the Modřany church | 50°00'18.6'' | 14°24'33.3'' | 26/09/2012 |
| 36 | *Oxalis stricta* | Černolice | Praha-západ | road edge at the summerhouse area, 1.7 km SSE of the railway station Dobřichovice | 49°54'47.4" | 14°17'59.1" | 18/07/2012 |
| 36 | *Oxalis stricta* | Praha-jesenice | Praha | road edge at Dolnocholupická street in the town | 49°59'58.3" | 14°24'38.8" | 27/07/2012 |
| 37 | *Oxybaphus nyctagineus* | Břeclav | Břeclav | railway bank at the railway station Břeclav-zastávka | 48°45'47.4'' | 16°54'10.4'' | 30/09/2012 |
| 38 | *Panicum capillare* | Týniště nad Orlicí | Rychnov nad Kněžnou | railway station Týniště nad Orlicí | 50°09'39.7'' | 16°04'07.0'' | 16/08/2012 |
| 38 | *Panicum capillare* | Kolín-Zálbí | Kolín | railway station Kolín Zálabí | 50°02'13.1'' | 15°12'36.8'' | 20/09/2012 |
| 39 | *Panicum miliaceum* | Drnholec | Břeclav | field edge, 1.8 km ESE of the Drnholec church | 48°51'14.0'' | 16°29'52.1'' | 29/09/2012 |
| 39 | *Panicum miliaceum* | Hrušky | Břeclav | field edge, 1.8 km WSW of the railway station Hrušky | 48°46'36.2'' | 16°55'12.7'' | 29/09/2012 |
| 40 | *Phytolacca esculenta* | Černošice | Praha-západ | alluvium of the Berounka river, 1.1 km NNE of the railway station Černošice | 49°58'00.8'' | 14°20'11.8'' | 25/09/2012 |
| 40 | *Phytolacca esculenta* | Břeclav | Břeclav | railway embankment near the railway station Břeclav-zastávka | 48°46'28.1'' | 16°54'48.9'' | 29/09/2012 |
| 41 | *Potentilla intermedia* | Lanžhot | Břeclav | railway station Lanžhot | 48°43'58.7'' | 16°57'41.2'' | 29/09/2012 |
| 41 | *Potentilla intermedia* | Týniště nad Orlicí | Rychnov nad Kněžnou | railway station Týniště nad Orlicí | 50°09'40.5'' | 16°04'03.4'' | 16/08/2012 |
| 42 | *Pseudofumaria lutea* | Průhonice | Praha-západ | low wall in the Park Průhonice | 50°00'02.1'' | 14°33'24.6'' | 08/08/2012 |
| 42 | *Pseudofumaria lutea* | Štramberk | Nový Jičín | garden walls in the street Zauličí | 49°35'14.8' | 18°07'23.7'' | 12/07/2012 |
| 43 | *Rudbeckia hirta* | Praha Modřany | Praha | ruderal lawn 0.6km NE of the Modřany church | 50°00'26.7'' | 14°24'53.7' | 26/09/2012 |
| 44 | *Rudbeckia laciniata* | Nasavrky | Chrudim | Chrudimka river bank at the summerhouse area, 1.7 km NNE of the Nasavrky church | 49°51'37.9' | 15°48'20.9'' | 24/09/2012 |
| 44 | *Rudbeckia laciniata* | Lanžhot | Břeclav | wet meadow, 2.7 km SSE of the railway station Lanžhot | 48°23'34.0'' | 16°58'24.9'' | 29/09/2012 |
| 45 | *Rumex alpinus* | Pec pod Sněžkou | Trutnov | road edge, 1.4 km NNW of the Pec pod Sněžkou church | 50°41'28.6'' | 15°42'54.1'' | 30/08/2012 |
| 45 | *Rumex alpinus* | Malá Úpa | Trutnov | road and meadow road edge, 0.6 km WSW of the municipality Malá Úpa village | 50°44'09.9'' | 15°47'54.3'' | 30/08/2012 |
| 46 | *Rumex longifolius* | Pec pod Sněžkou | Trutnov | road edge, 1.4 km NNW of the Pec pod Sněžkou church | 50°41'28.6'' | 15°42'54.1'' | 30/08/2012 |
| 46 | *Rumex longifolius* | Malá Úpa- Pomezní Boudy | Trutnov | road and meadow edge, 2.7 km NNE of the Malá Úpa church | 50°44'40.5'' | 15°49'22.6'' | 30/08/2012 |
| 47 | *Rumex patientia* | Pavlov | Břeclav | road bank, 1 km ESE of the Pavlov church | 48°52'14.3'' | 16°41'02.5'' | 15/07/2012 |
| 48 | *Rumex thyrsiflorus* | Velký Osek | Kolín | meadow, 2.7 km WNW of the railway station Velký Osek | 50°06'30.6'' | 15°09'26.9'' | 04/09/2012 |
| 48 | *Rumex thyrsiflorus* | Lanžhot | Břeclav | road edge, 3 km WNW of the railway station Lanžhot | 48°44'32.1'' | 16°56'13.4'' | 15/07/2012 |
| 48 | *Rumex thyrsiflorus* | Týniště nad Orlicí | Rychnov nad Kněžnou | railway station Týniště nad Orlicí | 50°09'37.4" | 16°04'03.4" | 16/08/2012 |
| 49 | *Scutellaria altissima* | Měšice u Prahy | Praha-východ | forest at the N edge of the Park Měšice | 50°11'47.6'' | 14°31'12.8'' | 08/07/2012 |
| 49 | *Scutellaria altissima* | Srbsko | Beroun | forest edge, 2 km ESE of the railway station Srbsko | 49°55'55.5'' | 14°09'23.2'' | 03/07/2012 |
| 49 | *Scutellaria altissima* | Roztoky-Tiché údolí | Praha-západ | forest edge closed to Unětický potok brook, 0,8 km WEW of the railway station Roztoky | 50°09'10.9'' | 14°23'21.4'' | 10/07/2012 |
| 50 | *Senecio inaequidens* | Sedlec-Praha | Praha | railway bank, 1 km S of the railway station Praha-Sedlec | 50°07'21.7" | 14°23'38.0" | 09/10/2012 |
| 50 | *Senecio inaequidens* | Praha Podbaba | Praha | railway bank, 1.4 km N of the railway station Praha-Podbaba | 50°07'26.7" | 14°23'47.1" | 09/10/2012 |

**Supplementary Table S5.** Continued

| **species ID** | **Species** | **locality** | **district** | **locality description** | **latitude** | **longitude** | **date of collection** |
| --- | --- | --- | --- | --- | --- | --- | --- |
| 51 | *Senecio leucanthemifolius subsp. vernalis* | Veltrusy | Mělník | sandpit 2.5 km SE of the Veltrusy church | 50°15'37.8'' | 14°20'27.0'' | 31/05/2012 |
| 51 | *Senecio leucanthemifolius subsp. vernalis* | Píšťany | Litoměřice | Žernosecké jezero sandpit, 0.8 km SW of the railway station Velké Žernoseky | 50°31'12.2'' | 14°03'55.4'' | 31/05/2012 |
| 52 | *Setaria faberi* | Kolín-Záblatí | Kolín | railway station Kolín-Zálabí | 50°02'13.1'' | 15°12'36.8'' | 20/09/2012 |
| 53 | *Sisymbrium loeselii* | Lanžhot | Břeclav | road edge, 1.3 km WSW of the railway station Lanžhot | 48°43'32.8'' | 16°56'57.9'' | 01/10/2012 |
| 53 | *Sisymbrium loeselii* | Pečky | Kolín | railway station Pečky | 50°05'29.5'' | 15°01'13.0'' | 06/09/2012 |
| 53 | *Sisymbrium loeselii* | Praha-Komořany | Praha | railway, 0.3 km S of the railway station Praha-Komořany | 50°00'09.3'' | 14°24'11.3'' | 24/08/2012 |
| 54 | *Sisymbrium strictissimum* | Strakonice | Strakonice | railway bank, 0.2 km S of the Strakonice castle | 49°15'21.3" | 13°54'04.3" | 25/09/2012 |
| 54 | *Sisymbrium strictissimum* | Praha-Komořany | Praha | road edge, 0.3 km S of the railway station Praha-Komořany | 49°59'39.8" | 14°24'20.1" | 26/09/2012 |
| 54 | *Sisymbrium strictissimum* | Hnanice | Znojmo | edge of the road, 1.5 km NNW of the Hnanice church | 48°48'42.2" | 15°58'40.8" | 29/09/2012 |
| 55 | *Smyrnium perfoliatum* | Praha-Zbraslav | Praha | forest edge, 0.7km SE of the railway station Praha-Zbraslav | 49°58'03.0'' | 14°24'27.8'' | 21/08/2012 |
| 55 | *Smyrnium perfoliatum* | Praha-Petřín | Praha | forest edge, 0.2km WSW of the lower funicular station | 50°04'57.2'' | 14°24'03.1'' | 29/07/2012 |
| 56 | *Solidago canadensis* | Praha-Komořany | Praha | road edge, 0.3 km NNE of the railway station Komořany | 49°58'02.3'' | 14°24'30.4'' | 26/09/2012 |
| 56 | *Solidago canadensis* | Pečky | Kolín | railway stationPečky | 50°05'25.5'' | 15°01'41.2'' | 05/10/2012 |
| 56 | *Solidago canadensis* | Lanžhot | Břeclav | ruderal site close to soil waste site, 1.4 km SE of the village | 48°43'35.0'' | 16°58'42.7'' | 01/10/2012 |
| 57 | *Solidago gigantea* | Mutěnice | Hodonín | railway bank, 0.6 km ENE of the railway station Mutěnice | 48°54'17.8'' | 17°02'59.6'' | 01/10/2012 |
| 57 | *Solidago gigantea* | Liteň | Beroun | railway station Liteň | 49°54'12.6'' | 14°09'15.9'' | 11/10/2012 |
| 58 | *Telekia speciosa* | Průhonice | Praha-západ | forest edge in the Park Průhonice | 49°59'49.3'' | 14°33'50.3'' | 14/09/2012 |
| 59 | *Xanthium albinum* | Břeclav Pohansko | Břeclav | riverside of Dyje river | 48°43'31.8'' | 16°53'26.9'' | 01/10/2012 |
| 59 | *Xanthium albinum* | Hrušky | Břeclav | sandpit bank, 4.2 km WSW of the railway station Hrušky | 48°46'37.9'' | 16°55'31.9'' | 01/10/2012 |
| 59 | *Xanthium albinum* | Píšťany | Litoměřice | Žernosecké jezero sandpit bank, 0.8 km SW of the railway station Velké Žernoseky | 50°31'12.5'' | 14°03'59.5'' | 06/10/2012 |

**Supplementary Fig. S1** Mean monthly soil temperature (green) and moisture (blue) recorded over the duration of the seed burial experiment between 2013 and 2020 at the common garden facility of the Institute of Botany, Průhonice, Czech Republic.
